# Collagen type VI regulates TGF-**β** bioavailability in skeletal muscle in mice

**DOI:** 10.1172/JCI173354

**Published:** 2025-05-01

**Authors:** Payam Mohassel, Hailey Hearn, Jachinta Rooney, Yaqun Zou, Kory Johnson, Gina Norato, Matthew A. Nalls, Pomi Yun, Tracy Ogata, Sarah Silverstein, David A. Sleboda, Thomas J. Roberts, Daniel B. Rifkin, Carsten G. Bönnemann

**Affiliations:** 1 National Institute of Neurological Disorders and Stroke, Neuromuscular and Neurogenetic Disorders of Childhood Section, NIH, Bethesda, Maryland, USA.; 2 Department of Neurology, Johns Hopkins University School of Medicine, Baltimore, Maryland, USA.; 3 Bioinformatics Section, Intramural Information Technology & Bioinformatics Program, National Institute of Neurological Disorders and Stroke, and; 4 Clinical Trials Unit, National Institute of Neurological Disorders and Stroke, NIH, Bethesda, Maryland, USA.; 5 Department of Ecology and Evolutionary Biology, University of California, Irvine, Irvine, California, USA.; 6 Department of Ecology and Evolutionary Biology, Brown University, Providence, Rhode Island, USA.; 7 Department of Cell Biology, New York University School of Medicine, New York, New York, USA.

**Keywords:** Genetics, Muscle biology, Extracellular matrix, Genetic diseases, Growth factors

## Abstract

Collagen VI–related disorders (*COL6*-RDs) are a group of rare muscular dystrophies caused by pathogenic variants in collagen VI genes (*COL6A1*, *COL6A2*, and *COL6A3*). Collagen type VI is a heterotrimeric, microfibrillar component of the muscle extracellular matrix (ECM), predominantly secreted by resident fibroadipogenic precursor cells in skeletal muscle. The absence or mislocalization of collagen VI in the ECM underlies the noncell-autonomous dysfunction and dystrophic changes in skeletal muscle with a yet elusive direct mechanistic link between the ECM and myofiber dysfunction. Here, we conducted a comprehensive natural history and outcome study in a mouse model of *COL6*-RDs (*Col6a2^–/–^* mice) using standardized (TREAT-NMD) functional, histological, and physiological parameters. Notably, we identify a conspicuous dysregulation of the TGF-β pathway early in the disease process and propose that the collagen VI–deficient matrix is not capable of regulating the dynamic TGF-β bioavailability both at baseline and in response to muscle injury. Thus, we propose a new mechanism for pathogenesis of the disease that links the ECM regulation of TGF-β with downstream skeletal muscle abnormalities, paving the way for the development and validation of therapeutics that target this pathway.

## Abstract 2

A novel mouse model of collagen VI-related muscular dystrophy and a new disease pathomechanism linking the ECM regulation of TGFβ with downstream skeletal muscle abnormalities

## Introduction

Collagen VI–related disorders (*COL6*-RDs) are a group of muscular dystrophies that combine clinical features of connective tissue diseases and muscular dystrophy (1) and clinically manifest with progressive skeletal muscle weakness and fibrosis, joint contractures, skin abnormalities, osteopenia, and respiratory insufficiency (2, 3). While progress has been made in understanding the underlying molecular mechanisms of *COL6*-RDs, no approved disease-modifying therapies are available, and treatment relies on symptomatic management.

Mild and non-progressive muscle weakness is common in genetic connective tissue diseases — for example, Marfan’s syndrome due to *FBN1* pathogenic variants (4), osteogenesis imperfecta due to *COL1A1* pathogenic variants (5), and Ehlers-Danlos/myopathy overlap due to *COL12A1* pathogenic variants (6). However, muscle weakness in patients with *COL6*-RDs has a progressive and degenerative course characterized by dystrophic changes and muscle fibrosis.

Collagen type VI is a microfibrillar component of the extracellular matrix (ECM), with suprastructural similarities to fibrillin microfibrils, with which it often copurifies (7, 8). Its expression is widespread in brain, blood vessels, skin, cardiac and skeletal muscle, and connective tissues (for example, cartilage, bone, tendon, ligaments, and joints), as well as cornea and placenta (1). Collagen VI is assembled intracellularly from three α chains encoded by *COL6A1*, *COL6A2*, and *COL6A3*. Three other genes (*COL6A4*, a pseudogene in humans; *COL6A5*; and *COL6A6*) share homology with *COL6A3* (9, 10); however, no pathogenic variants in these genes leading to *COL6*-RDs have been identified. The collagen VI α1, α2, and α3 chains each contain a short triple helical domain and assemble from the C-terminal to the N-terminal direction into a heterotrimeric monomer. Heterotrimeric monomers form antiparallel dimers, which subsequently assemble into tetramers before secretion into the extracellular space, where they join to form a microfibrillar structure in the skeletal muscle basement membrane. Variants that cause the absence of any of the essential α chains (typically biallelic) or result in assembly-competent mutant chains (typically monoallelic) most often underlie the disease (11).

How abnormalities in collagen VI in the muscle ECM lead to muscular dystrophy remains incompletely understood. Mechanisms of disease in other muscular dystrophies such as sarcolemmal fragility, impaired membrane repair, abnormalities in muscle enzymes, intermediate filaments or nuclear envelope proteins, or myofibrillar or Z disk–associated proteins (12, 13) have not been shown to be the primary drivers of disease in *COL6-*RDs. Serum creatine kinase levels are consistently normal or only mildly elevated in *COL6-*RD patients (14). In addition, collagen VI is predominantly produced and secreted by skeletal muscle interstitial fibroblasts and fibroadipogenic precursor cells and not muscle fibers themselves (15), which further adds to the complexities of *COL6-*RD pathogenesis.

A few mouse models of *COL6*-RDs have been generated and characterized so far: *Col6a1*-deficient mice (16, 17), *Col6a3*-deficient mice (18), and 2 dominant-negative mutant mouse models with deletion of exon 16 of *Col6a3* or exon 5 of *Col6a2* (19, 20) (Supplemental Table 1; supplemental material available online with this article; https://doi.org/10.1172/JCI173354DS1). These mouse models show variable myopathic changes and abnormalities, milder than those in *COL6*-RD patients with analogous pathogenic variants. In the *Col6a1*-knockout mice (16), a few downstream cell physiological disturbances in skeletal muscle, including reduction of autophagy flux (21), mitochondrial abnormalities and apoptosis (22), and reduced satellite cell regenerative reserve (17, 23), have been reported. However, the underlying mechanism of how collagen VI abnormalities in the muscle ECM lead to these downstream cellular abnormalities and muscular dystrophy has not been fully delineated.

To add to the models of the disease and better understand the underlying mechanisms of *COL6*-RD, we derived and characterized a mouse model, *Col6a2^–/–^* mice, from the UC Davis Knockout Mouse Project. To establish a robust preclinical model for mechanistic and therapeutic studies, we have comprehensively characterized the natural history of disease in this mouse model using behavioral, physiological, and histological studies of skeletal muscle. We identify a conspicuous dysregulation of the TGF-β pathway early in the disease process and propose that the collagen VI–deficient matrix is not capable of dynamic regulation of TGF-β bioavailability and thus propose a new mechanism for pathogenesis of the disease that links the ECM with downstream skeletal muscle abnormalities and fibrosis.

## Results

### Col6a2^–/–^ mice develop early postnatal muscle atrophy and weakness.

**Figure 1:**
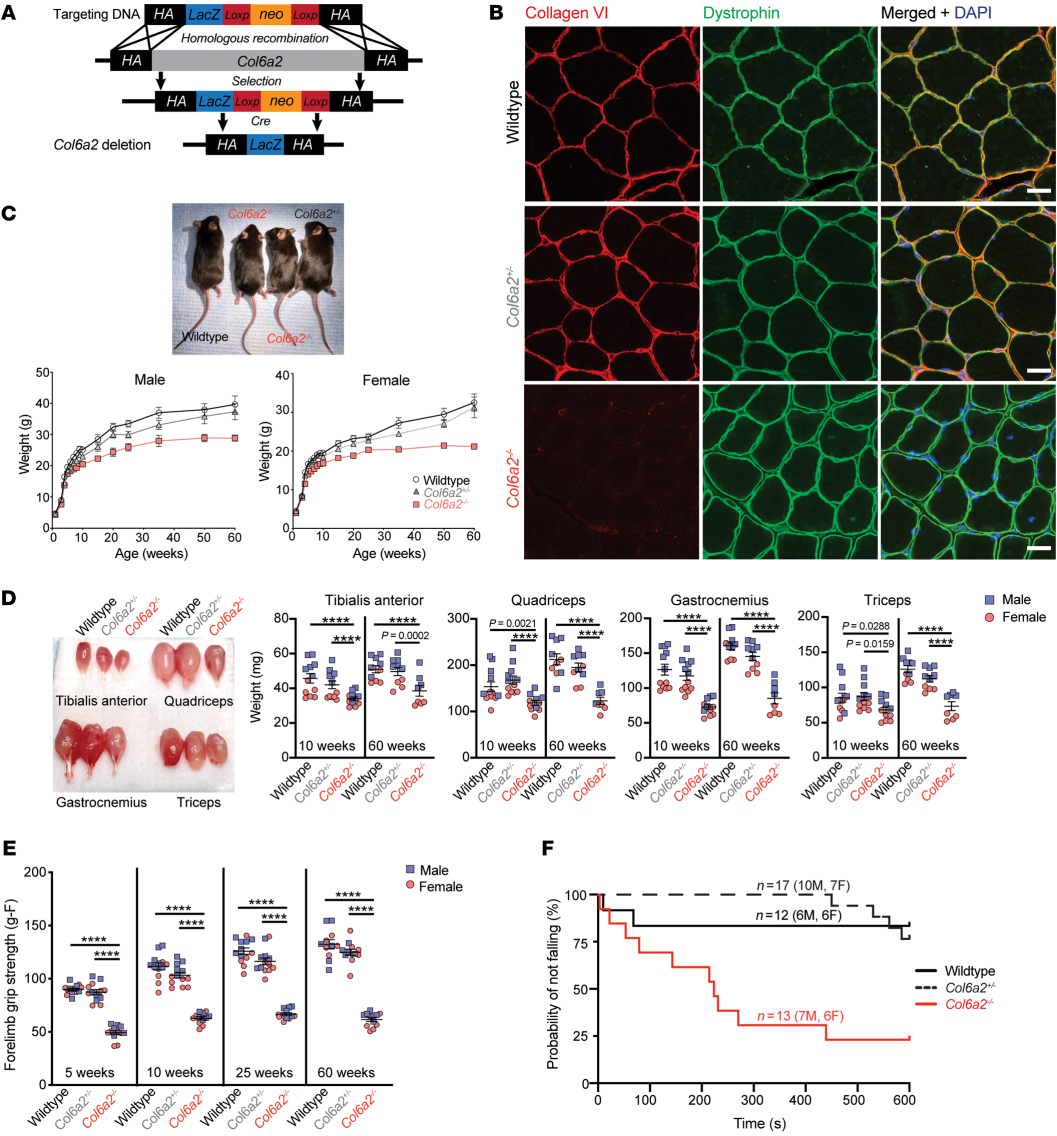
*Col6a2^–/–^* mice develop early postnatal muscle atrophy and weakness. (**A**) Schematic representation of the *Col6a2* knockout allele. The *LacZ* sequence is not expressed but is used in the genotyping strategy. (**B**) Immunofluorescence staining of frozen gastrocnemius muscle shows complete absence of collagen VI from the muscle ECM in *Col6a2^–/–^* mice. Scale bars: 25 μm. (**C**) *Col6a2^–/–^* mice weigh less than WT and *Col6a2^+/–^* littermates. (**D**) Male and female *Col6a2^–/–^* mice have smaller muscles compared with WT and *Col6a2^+/–^* littermates. (**E**) Male and female *Col6a2^–/–^* mice have markedly reduced forelimb grip strength compared with WT and *Col6a2^+/–^* littermates. (**F**) *Col6a2^–/–^* mice have an increased probability of falling in the hanging wire test (10 weeks old). Statistical comparisons in **F** were performed using Cox proportional hazard model. Genotype is significantly related to the hazard of falling (log-rank test = 17.9, df = 2; *P* < 0.001), with the following pairwise differences: WT vs. *Col6a2^–/–^*, HR = 0.12 (95% CI: 0.03–0.57), *P* = 0.02; *Col6a2^+/–^* vs. *Col6a2^–/–^*, HR = 0.15 (95% CI: 0.05–0.50), *P* = 0.006; *Col6a2^+/–^* vs. WT, HR = 1.25 (95% CI: 0.23–6.84), *P* = 1.0. M, male; F, female. For all other panels, error bars indicate SEM, and statistical comparisons were performed by 2-way ANOVA with Tukey’s adjustment for multiple comparisons. ****P < 0.0001.

We obtained and backcrossed a previously uncharacterized mouse strain with complete deletion of the *Col6a2* genomic locus from the UC Davis Knockout Mouse Project Repository (Figure 1A). Unlike WT and heterozygous (*Col6a2^+/–^*) littermates, mice with homozygous deletion of Col6a2 (*Col6a2^–/–^*) had complete absence of collagen VI staining in skeletal muscle ECM (Figure 1B). *Col6a2^–/–^* mice weighed less and appeared smaller than littermates (Figure 1C), a difference that was first noted at around 4 weeks of age. They had a normal lifespan and were present in slightly reduced Mendelian ratios (20.6%) (Supplemental Figure 1). Isolated *Col6a2^–/–^* skeletal muscles weighed less than those of littermates and remained proportionally atrophic in mice as old as 60 weeks (Figure 1D).

*Col6a2^–/–^* mice had markedly reduced forelimb grip strength detected as early as 5 weeks of age through 60 weeks of age compared with controls (Figure 1E) and were more likely to fall in the hanging wire test assessed at 10 weeks of age (Figure 1F). Other functional tests such as rotarod, treadmill running, and activity monitoring were highly variable and failed to identify a difference between *Col6a2^–/–^* mice and littermate controls (Supplemental Figure 1).

### Col6a2^–/–^ skeletal muscles show mild dystrophic features.

**Figure 2:**
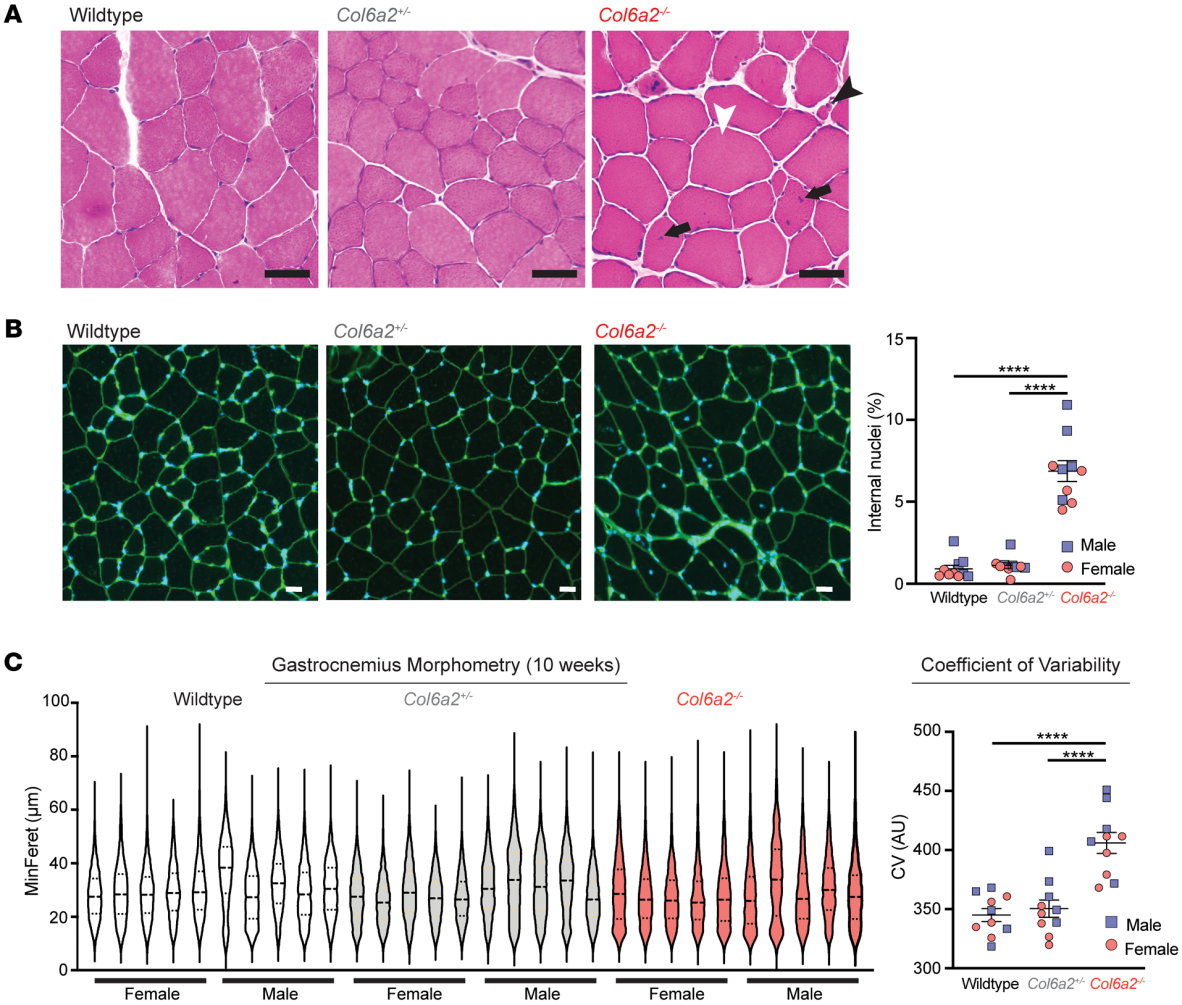
*Col6a2^–/–^* muscle shows myofiber atrophy and dystrophic changes. (**A**) Hematoxylin and eosin (H&E) staining of tibialis anterior frozen sections (10 weeks old) shows dystrophic features including fiber size variability with myofiber atrophy (black arrowhead), hypertrophy (white arrowhead), and increased internal nuclei (arrows). Scale bars: 50 μm. (**B**) Wheat germ agglutinin–stained (green) and DAPI-stained gastrocnemius muscle (10 weeks old). Percentage of fibers with internal nuclei was markedly increased in *Col6a2^–/–^* muscle. Scale bars: 50 μm. (**C**) The minimum Feret diameter (MinFeret) of all myofibers in the entire gastrocnemius muscle sections (10 weeks old) was reduced in *Col6a2^–/–^* mouse muscle with a marked increase in fiber size variability measured by coefficient of variability (CV). AU, arbitrary units. Error bars indicate SEM. Statistical comparisons were performed by 2-way ANOVA and Tukey’s adjustment for multiple comparisons. ****P < 0.0001.

*Col6a2^–/–^* mouse muscle showed dystrophic histological features including fiber size variability with atrophic and hypertrophic myofibers and increased endomysial fibrosis (Figure 2A). Similar findings including rare degenerating and regenerating myofibers were also observed in gastrocnemius, quadriceps, triceps, and diaphragm muscles (Supplemental Figure 2). Immunostaining for specific myosin subtypes failed to identify selective loss or atrophy of oxidative or glycolytic myofibers (Supplemental Figure 2). There was a substantial increase in internal nuclei in *Col6a2^–/–^* muscle, an indirect marker of myofiber regeneration (Figure 2B). Minimal Feret diameter of myofibers in gastrocnemius muscle sections was quantitatively assessed using Myosoft open-source software (24). In 10-week-old animals, fiber size variation, measured by coefficient of variability (standard deviation of minimal Feret diameter × 1,000/mean of minimal Feret diameter), was markedly increased in *Col6a2^–/–^* muscle compared with heterozygous and WT controls (Figure 2C). Similar findings were observed in 60-week-old animals (Supplemental Figure 2).

### Col6a2^–/–^ skeletal muscle produces reduced contractile force and is susceptible to lengthening contraction injury.

**Figure 3:**
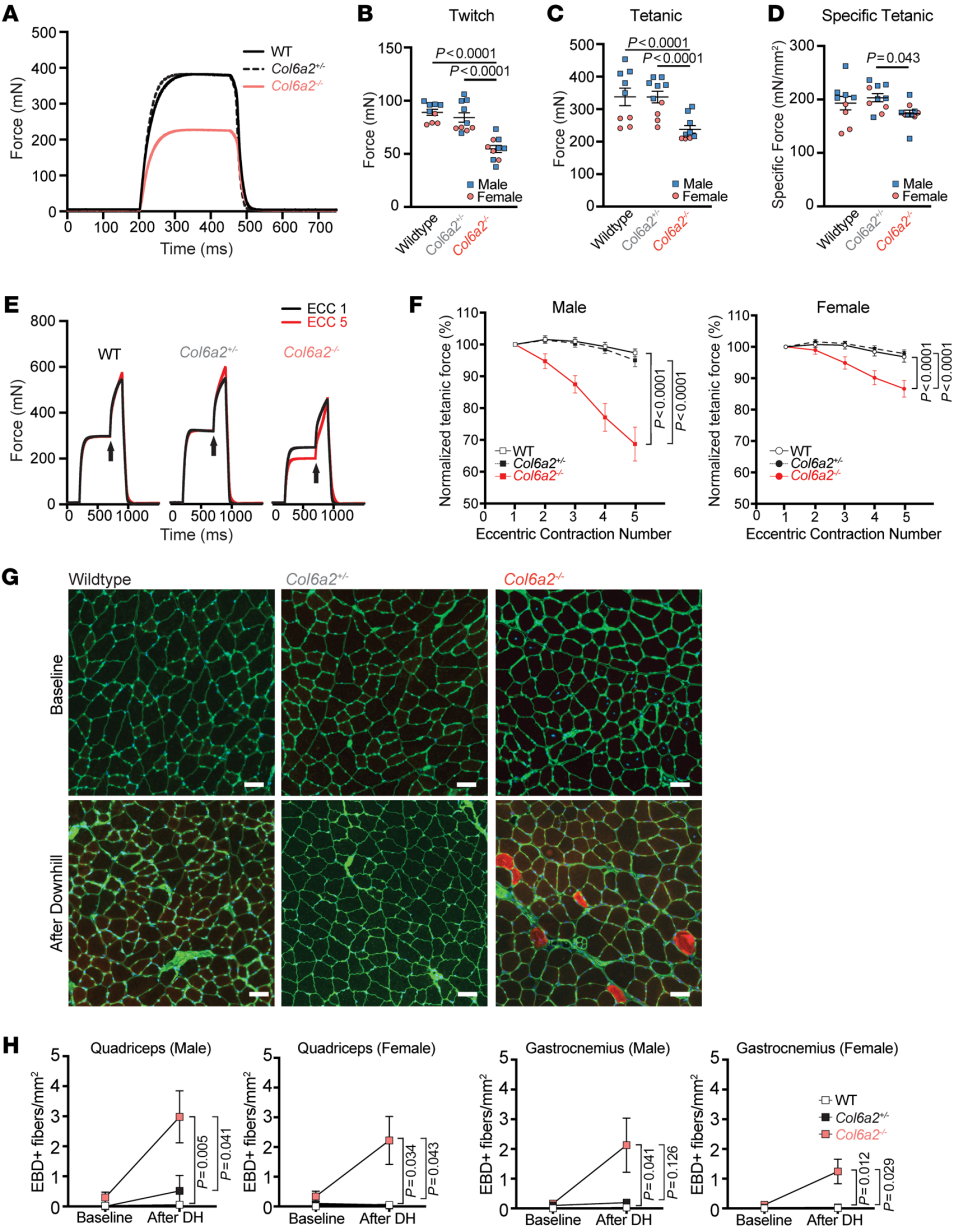
*Col6a2^–/–^* muscle has reduced contractile force and is susceptible to lengthening contraction injury. (**A**) Representative tracing of total tetanic force measurement in isolated EDL muscle. (**B** and **C**) Maximal twitch force (**B**) and total tetanic force (**C**) are markedly reduced in 10-week-old *Col6a2^–/–^* mouse EDL muscle. (**D**) The difference in force generation was negligible after normalization to functional cross-sectional area (i.e., specific force). (**B**–**D**) Error bars indicate SEM. Statistical comparisons were performed by 2-way ANOVA and Tukey’s adjustment for multiple comparisons. (**E**) Representative tracing of force measurement in isolated EDL muscle after eccentric contraction. (**F**) Tetanic force declined precipitously after repeated eccentric contractions in *Col6a2^–/–^* EDL muscle. The decline was more prominent in male animals (*n* = 5 male, 5 female for WT and *Col6a2^+/–^*, and *n* = 8 male, 5 female for *Col6a2^–/–^*). Statistical analysis was performed using linear mixed models and Bonferroni adjustment for multiple comparisons. (**G**) Muscle cryosections stained with fluorescent wheat germ agglutinin (green) and DAPI (blue) after downhill running and systemic administration of Evans blue dye (EBD). Red fibers (EBD positive) represent those with damaged sarcolemma. Scale bars: 50 μm. (**H**) Quantification of EBD-positive fibers normalized to muscle area. For statistical comparisons, ordinary 1-way ANOVA was performed at baseline (no differences) and after downhill running with Bonferroni’s adjustment for multiple comparisons. WT, *n* = 13 (7 male, 6 female); *Col6a2^+/–^*, *n* = 9 (4 male, 5 female); *Col6a2^–/–^*, *n* = 13 (6 male, 7 female). Error bars in all panels represent SEM.

Physiological parameters in a standard ex vivo preparation of isolated extensor digitorum longus (EDL) muscle in 10-week-old animals showed a decrease in twitch and maximum tetanic force in *Col6a2^–/–^* muscle (Figure 3, A–C). However, specific force, i.e., tetanic force normalized to functional cross-sectional area of the muscle, was only minimally reduced (Figure 3D). We also performed repeated eccentric contractions (10% of optimal length), which resulted in a marked reduction of the tetanic force in *Col6a2^–/–^* muscle, suggestive of its susceptibility to eccentric contraction injury (Figure 3E). Notably, female *Col6a2^–/–^* muscle showed a less dramatic reduction in tetanic force after repeated eccentric contractions in comparison with male animals (Figure 3F). Similar contractile force and physiological parameters were noted in 60-week-old animals, and 60-week-old female *Col6a2^–/–^* mice were also protected against tetanic force reduction after repeated eccentric contractions (Supplemental Figure 3). To investigate the susceptibility to lengthening contraction injury in vivo, we used a downhill treadmill running paradigm followed by Evans blue dye injection. Consistent with the ex vivo physiology testing, we found an increase in sarcolemmal fragility and Evans blue dye uptake in *Col6a2^–/–^* muscle fibers after downhill running (Figure 3, G and H), though without an apparent protection in female animals.

### Col6a2^–/–^ muscle ECM is fibrotic, has abnormal morphology, and shows impaired passive mechanical properties.

**Figure 4:**
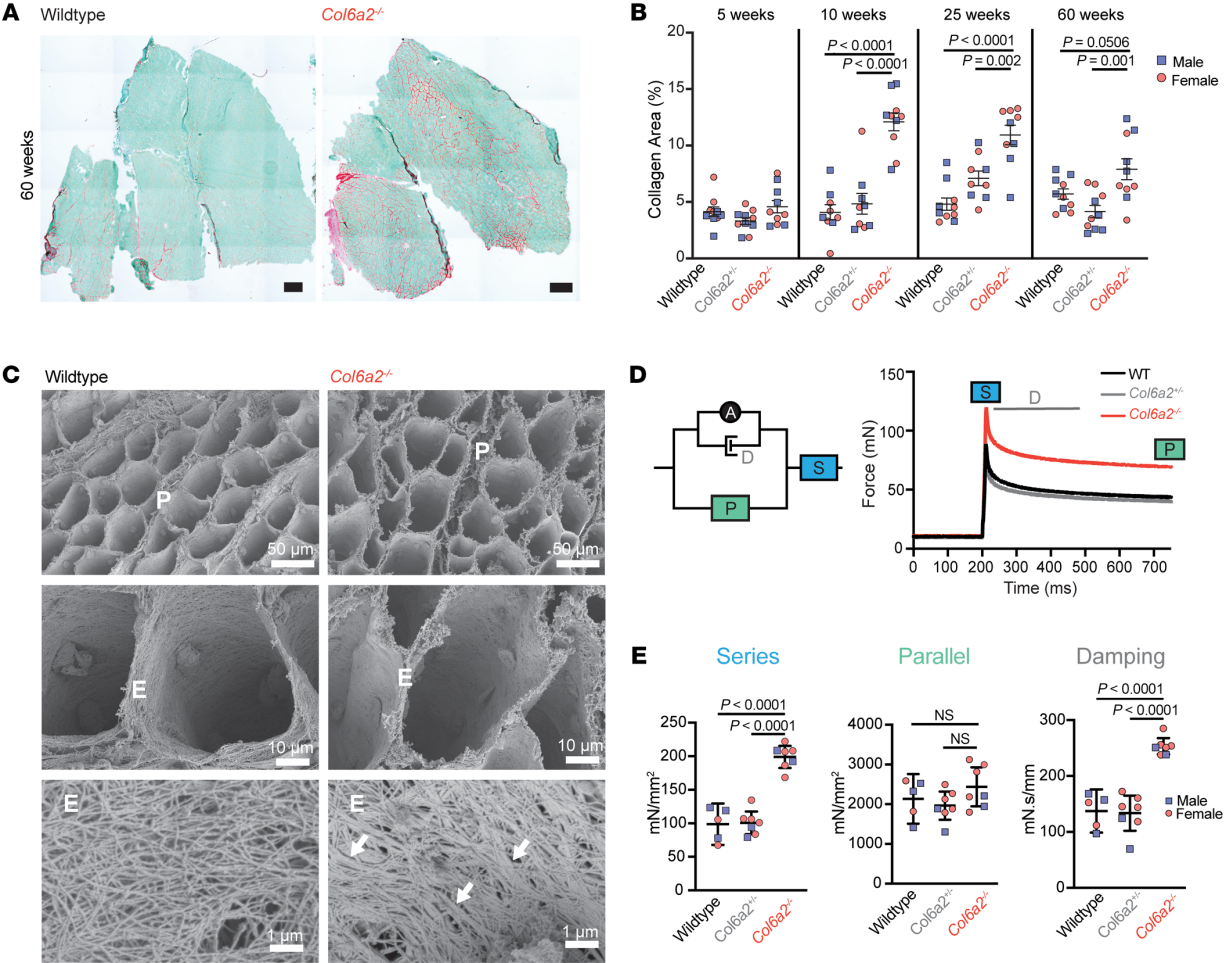
*Col6a2^–/–^* muscle ECM is fibrotic, has abnormal morphology, and shows altered passive mechanical properties. (**A**) Representative image of Sirius red stain shows increased fibrillar collagen content (collagen I and collagen III) in *Col6a2^–/–^* mouse muscle. Scale bars: 500 μm. (**B**) Quantification of the Sirius red stain (collagen area) in gastrocnemius muscle sections shows marked increase in fibrillar collagen in mice 10 weeks of age or older. (**C**) Scanning electron microscope images of decellularized lateral gastrocnemius muscle of WT and *Col6a2^–/–^* mouse show altered ECM morphology, increased thickness, and variability of fibrillar collagen most notably in the endomysium (arrows). Myonuclear impressions on the endomysium are noted in the top and middle panels. P, perimysium; E, endomysium. (**D**) Schematic of A.V. Hill viscoelastic mechanical model of skeletal muscle. The right panel shows the force tracing of isolated EDL muscle after passive stretch. The different components of the model are calculated from different portions of the stress-relaxation protocol as indicated. S, series elastic element; P, parallel elastic element; D, damping; A, active contractile element. (**E**) Quantification of passive mechanical properties of isolated EDL muscle in 60-week-old animals shows a marked increase in series modulus of elasticity and damping coefficient, but not the parallel elastic element, in *Col6a2^–/–^* mice. Statistical comparisons were performed by 2-way ANOVA and Tukey’s adjustment for multiple comparisons. Error bars represent SEM.

Muscle ECM remodeling and fibrosis are pathological hallmarks of muscular dystrophies, including *COL6*-RDs. We found a marked increase in endomysial fibrillar collagen content based on the Sirius red staining and hydroxyproline content of gastrocnemius muscle of 25-week-old or older *Col6a2^–/–^* mice (Figure 4, A and B, and Supplemental Figure 4). Similar results were found in the diaphragm muscle, with markedly increased hydroxyproline content in *Col6a2^–/–^*– mice (Supplemental Figure 5). However, *Col6a2^–/–^* muscle at 5 weeks of age — a time point when these mice show reduced grip strength, muscle atrophy, and other dystrophic histological features — was not more fibrotic. Scanning electron microscopic evaluation of collagen fibers in decellularized lateral gastrocnemius muscles in *Col6a2^–/–^* mice showed an increase in thickness and variability in orientation of collagen fibers in the endomysium and perimysium, consistent with increased fibrillar collagen deposition and disorganization of the muscle ECM (Figure 4C).

To assess the functional effects of fibrosis and ECM remodeling on passive mechanical properties of *Col6a2^–/–^* skeletal muscle, we adapted an experimental paradigm to test viscoelastic properties of EDL muscle (25). Physiological components of this viscoelastic model reflect the contribution of muscle tissue components, including ECM, myotendinous junction, sarcolemma, and myofibril-related proteins (e.g., titin) (26). We found a marked increase in the series modulus of elasticity and damping, while the parallel modulus of elasticity of the model was unchanged (Figure 4, D and E, and Supplemental Figure 4C). The muscle tissue component contributing to both the series modulus of elasticity and damping is likely the ECM. These findings thus reflect the abnormal ECM morphology in *Col6a2^–/–^* skeletal muscle and suggest that inefficient force transmission in the ECM may contribute to muscle weakness in *Col6a2^–/–^* muscle.

### TGF-β pathway is upregulated in Col6a2^–/–^ skeletal muscle.

**Figure 5:**
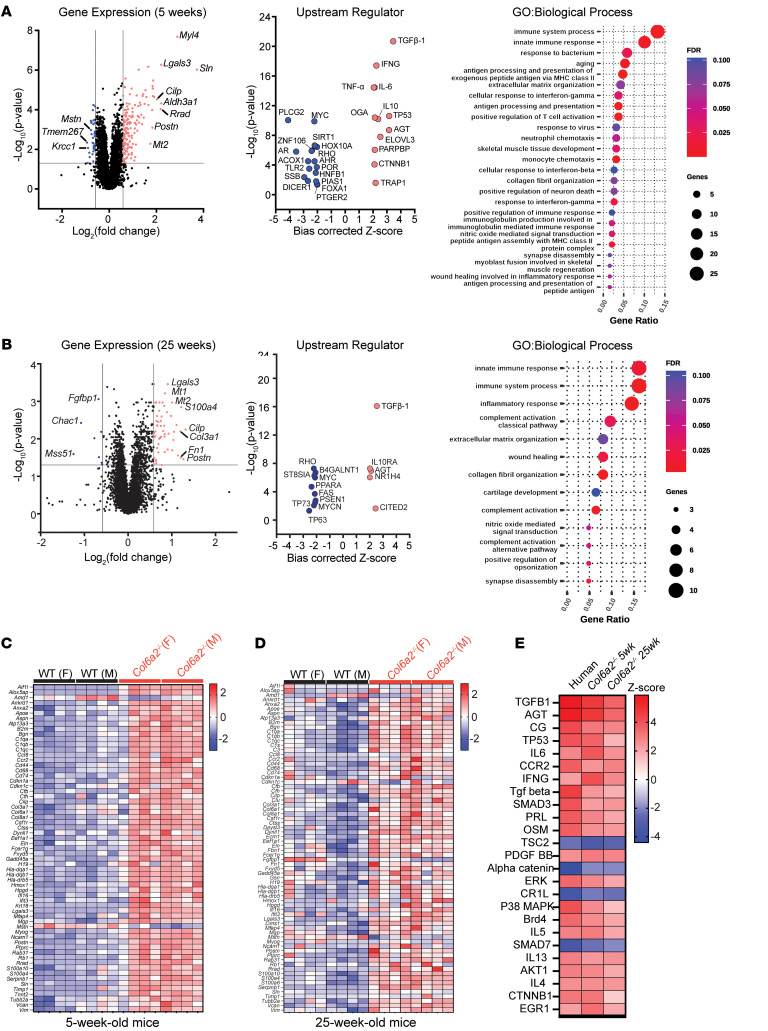
RNA-Seq identifies a robust upregulation of TGF-β pathway–related genes in *Col6a2^–/–^* mouse muscle. (**A**) Volcano plot of differentially regulated transcripts in 5-week-old *Col6a2^–/–^* quadriceps muscle RNA-Seq dataset. Upstream regulator analysis of differentially regulated transcripts identified the TGF-β pathway followed by immune system and cytokine-related pathways. Gene Ontology analysis identified immune system processes and collagen fibril and extracellular matrix–related terms. (**B**) RNA-Seq from 25-week-old mice shows results similar to those in **A**, again with marked upregulation of the TGF-β pathway in the upstream regulator analysis. (**C** and **D**) Heatmap representation of TGF-β–related transcripts depicts the dysregulated transcripts that underlie the identification of the TGF-β pathway as an upstream regulator. (**E**) Comparison analysis of RNA-Seq data from the *COL6-*RD human muscle biopsy study (28) and 5-week-old and 25-week-old *Col6a2^–/–^* mouse muscle compared with controls. Note upregulation of the TGFB1 pathway in all 3 datasets.

To evaluate the underlying molecular mechanisms of disease in *Col6a2^–/–^* mice, we performed a comprehensive RNA-Seq study of muscle tissue in juvenile (5-week-old) and adult (25-week-old) mice compared with WT littermate controls (*n* = 8, 4 male, 4 female, at each age group per genotype). In the juvenile mice, several ECM- and muscle-related transcripts (for example, *Myl4*, *Lgals3*, *Sln*, *Postn*, *Cilp*, *Fn*) were upregulated (Figure 5A and Supplemental Data File 1). In addition, *Mstn*, the gene encoding myostatin, was downregulated in *Col6a2^–/–^* muscle, a finding consistent with other models of muscular dystrophy (27). To evaluate the master regulators of the observed transcriptomic signatures, we used the Upstream Regulator Analysis bioinformatics tool (Ingenuity Pathway Analysis [IPA], QIAGEN), which showed a marked upregulation of the TGF-β pathway and other immune system/cytokine-related pathways such as interferon-γ and IL-6, among others (Figure 5A). Gene Ontology analysis of dysregulated genes using the DAVID database also identified immune system pathways as well as biological processes related to collagen fiber organization and ECM. Similar findings were noted in 25-week-old animals, again with the TGF-β pathway as the most upregulated upstream regulator (Figure 5B). To scrutinize the apparent upregulation of the TGF-β pathway as a master regulator, we generated heatmaps of the expression profile of more than 70 TGF-β pathway–related genes in each individual sample in the dataset (Figure 5, C and D). These results were consistent with a robust dysregulation of TGF-β–related genes in *Col6a2^–/–^* muscle, the vast majority of which were upregulated across the different samples without sex-specific differences.

We have recently reported the upregulation of the TGF-β pathway in muscle biopsies of patients with *COL6*-RDs (28). To compare the transcriptomic profile of muscle tissue in our mouse model with that in human biopsies, we performed a comparison study of upstream regulators in these datasets using the IPA bioinformatics tools. Upstream regulators and pathways with the most dramatic upregulation or downregulation were common between the human and the *Col6a2^–/–^* mouse datasets (Figure 5E and Supplemental Data File 1).

**Figure 6:**
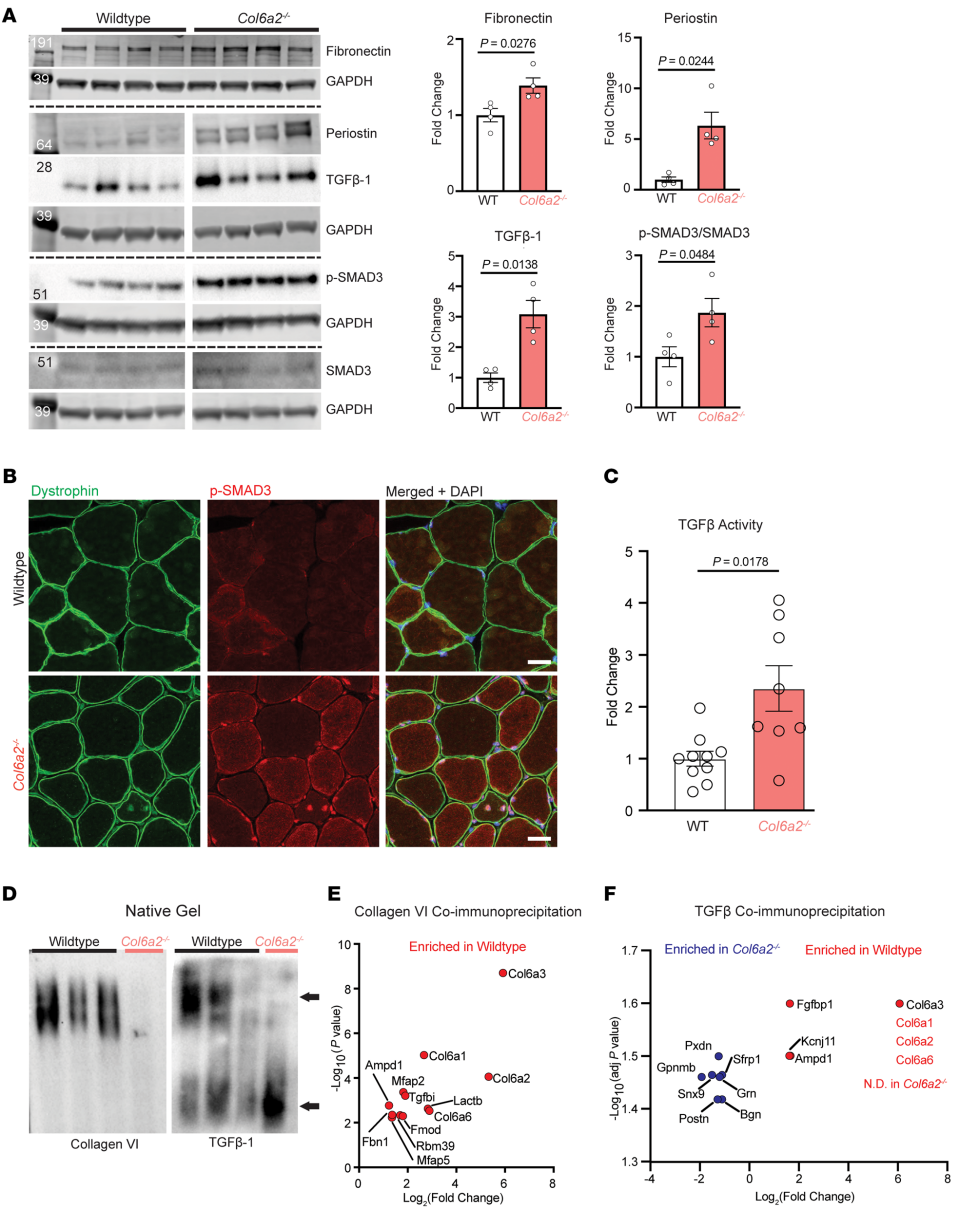
Active TGF-β is increased in resting *Col6a2^–/–^* muscle. (**A**) Western blotting of mouse muscle lysates under reducing and denaturing conditions shows an increase in TGF-β1 and its downstream effectors in *Col6a2^–/–^* muscle lysates. Corresponding bands were quantified and normalized to GAPDH as an internal control and graphed as fold change in comparison with WT. Error bars represent SEM. For statistical comparisons, a parametric, 2-tailed, unpaired *t* test with Welch’s correction was used (*n* = 4). (**B**) Immunofluorescence staining of muscle tissue shows increased p-SMAD3 staining in myonuclei, including internal nuclei of *Col6a2^–/–^* muscle. Scale bars: 25 μm. (**C**) HEK293-Luc TGF-β reporter assay shows that *Col6a2^–/–^* muscle fibroblast cultures deposit 2- to 3-fold higher levels of active TGF-β compared with WT controls in conditioned medium. Each data point is from an independent preparation (biological replicates) normalized to the average of WT samples for each run (WT, *n* = 10; *Col6a2^–/–^*, *n* = 8). (**D**) Blue native gel electrophoresis (not denatured, not reduced), followed by Western blotting of muscle lysates using anti–collagen VI or anti–TGF-β1 antibodies (as indicated). As expected, collagen VI tetramers are absent from *Col6a2^–/–^* muscle. TGF-β1 migrates in 2 different protein complexes (arrows), with the lower complex highly increased in *Col6a2^–/–^* muscle lysates compared with WT controls. (**E**) Collagen VI coimmunoprecipitation (using anti-Col6a1 antibody) of muscle lysates from WT and *Col6a2^–/–^* mice followed by quantitative data-independent acquisition (DIA) mass spectrometry identifies putative collagen VI binding partners in skeletal muscle (*n* = 5). (**F**) TGF-β coimmunoprecipitation (using anti-1D11 antibody) of muscle lysates followed by quantitative DIA mass spectrometry identifies different TGF-β binding partners in WT and *Col6a2^–/–^* skeletal muscle, corresponding to the different protein complexes identified in **D** (*n* = 4). Only proteins with an extracellular or plasma membrane localization are shown.

To validate the transcriptomics findings, we performed Western blotting of selected proteins, including fibronectin, periostin, and phosphorylated SMAD3 (p-SMAD3), as downstream intracellular effectors of the TGF-β pathway (Figure 6A). These proteins were markedly elevated in *Col6a2^–/–^* muscle lysates. In addition, p-SMAD3 expression was notably increased in myonuclei, including internally placed nuclei of regenerating fibers (Figure 6B).

### Absence of collagen VI from the ECM increases TGF-β activation.

TGF-β isoforms 1, 2, and 3 are three of the canonical TGF-β superfamily ligands that bind and activate TGF-β receptors, which in turn phosphorylate SMAD2/3, which, along with SMAD4, translocate to the cell nucleus to induce transcription of the TGF-β pathway–related genes. TGF-β ligands are normally sequestered in the ECM in a latent but poised protein complex and are released in response to specific environmental stimuli (29). Although the TGF-β pathway was markedly upregulated in *Col6a2^–/–^* mouse muscle, surprisingly, the total transcript levels of three TGF-β isoforms (TGF-β1, TGF-β2, and TGF-β3) were not increased (Supplemental Figure 6A). Total TGF-β1 protein levels measured by ELISA in muscle lysates were also unchanged (Supplemental Figure 6B). These data suggested that changes in TGF-β activation dynamics, instead of its overall transcription and expression, underlie TGF-β pathway overactivity in *Col6a2^–/–^* muscle.

To investigate this further, we first used Western blotting and found an increase in the abundance of active TGF-β1 protein (its 25 kDa dimer) in the soluble fraction of *Col6a2^–/–^* muscle lysates (Figure 6A). In addition, we measured the active TGF-β content deposited in conditioned medium of WT and *Col6a2^–/–^* muscle fibroblast cultures using a reporter HEK293 cell line that expresses firefly luciferase under the control of a TGF-β/p-SMAD2/3–responsive promoter (Supplemental Figure 6, C and D). These experiments showed a marked increase in TGF-β activity in *Col6a2^–/–^*-derived conditioned medium compared with controls (Figure 6C). Together, these findings suggest that absence of collagen VI from the muscle ECM increases active TGF-β without an increase in total TGF-β transcription or translation.

### Absence of collagen VI alters the TGF-β protein complex.

Microfibrillar collagen VI and fibrillin-1 share a similar suprastructure (7, 8). Since fibrillin-1 is known to bind the TGF-β–latent complex in the ECM and thus regulate its bioavailability (30–32), we hypothesized that collagen VI also binds the TGF-β–latent complex. We used native gel electrophoresis of muscle lysates to preserve protein-protein interactions followed by Western blotting to evaluate collagen VI– and TGF-β–associated protein complexes in muscle. We first confirmed the complete absence of the native collagen VI protein in *Col6a2^–/–^* muscle lysates (Figure 6D) and then assessed the TGF-β–associated protein complex in muscle lysates. This experiment showed a different pattern of migration, with the majority of TGF-β migrating in a lower–molecular weight protein complex, in *Col6a2^–/–^* muscle lysates compared with controls (Figure 6D). These data suggest that absence of collagen VI from the ECM alters the TGF-β protein-protein interactions.

To investigate the collagen VI and TGF-β protein-protein interactions in skeletal muscle further, we used a coimmunoprecipitation (co-IP) followed by data-independent acquisition (DIA) mass spectrometry paradigm. Using an anti–collagen VI antibody (Ab6588, raised against COL6A1) and the *Col6a2^–/–^* muscle as a negative control, we identified proteins enriched in the WT co-IP elutes (*n* = 5 per genotype). These results confirmed the well-known association of different collagen VI single chains with each other, all of which were highly enriched in the WT samples, as well as other TGF-β–regulating proteins, such as decorin, and microfibril-associated proteins Mfap2 (Magp-1) and Mfap5 (31, 33, 34). Several ECM proteins that we believe have not previously been reported to interact with collagen VI were also identified, including fibrillin-1 (a microfibrillar protein), Tgfbi (a protein related to periostin), and fibromodulin and lumican (proteoglycans) (Figure 6E and Supplemental Data File 2).

To investigate the different TGF-β–binding proteins in *Col6a2^–/–^* muscle, we performed co-IP/DIA mass spectrometry experiments using the anti–TGF-β antibody (1D11) in WT and *Col6a2^–/–^* muscle lysates (*n* = 4 per genotype). This antibody binds the active TGF-β dimer, allowing us to compare the TGF-β–binding proteins corresponding to the bands noted on native gel blots in Figure 6D. The most dramatically enriched proteins in the WT samples were collagen VI chains, suggesting a molecular complex that contains active TGF-β and collagen VI. TGF-β–binding proteins enriched in *Col6a2^–/–^* muscle included biglycan, a known TGF-β–binding protein, and other proteins that may regulate TGF-β activity (Figure 6E and Supplemental Data File 2). Overall, these data show that active TGF-β is in different protein complexes in the WT versus *Col6a2^–/–^* muscle, which likely contributes to the altered TGF-β activity in *Col6a2^–/–^* muscle.

### Col6a2^–/–^ muscle regeneration is delayed and its ECM components are altered after acute muscle injury.

**Figure 7:**
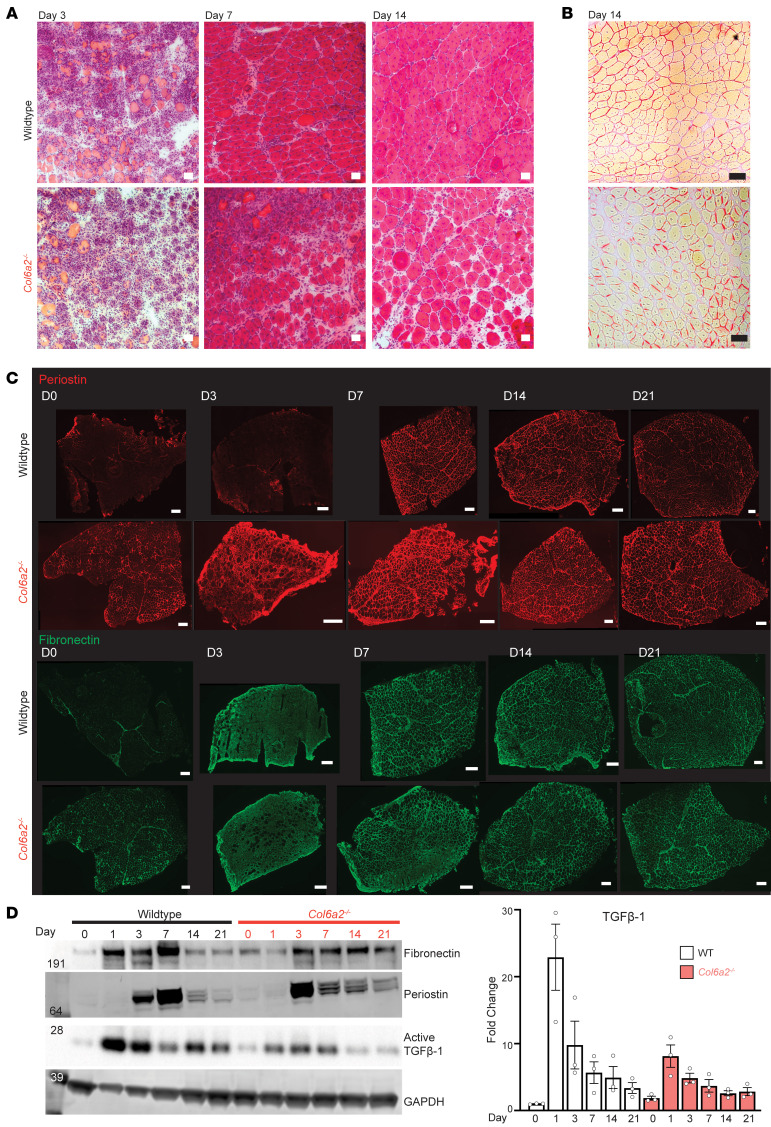
Collagen VI dampens TGF-β availability in acute phases of muscle regeneration after injury. (**A**) H&E staining of tibialis anterior muscle after CTX injury shows delayed regeneration in *Col6a2^–/–^* mice with persistent inflammatory infiltrates (day 7 and day 14), myofiber/myotube smallness, and increased endomysial spaces. Scale bars: 50 μm. (**B**) Sirius red stain of tibialis anterior muscle 14 days after CTX injury. Scale bars: 50 μm. (**C**) Immunostaining of tibialis anterior muscle sections with periostin and fibronectin at different time points after CTX injury. Scale bars: 200 μm (**D**) Representative Western blots of muscle lysates for TGF-β1, periostin, and fibronectin after CTX injury. Right panel: TGF-β band intensity was quantified and normalized to GAPDH as an internal control and compared with the WT day 0 (prior to CTX injury). Data points represent 3 biological replicates for each time point. Error bars represent SEM.

Previous studies of other *COL6*-RD mouse models have noted an inadequate muscle regenerative response to injury (17, 23). To assess the regenerative capacity of *Col6a2^–/–^* mouse muscle, we used the cardiotoxin (CTX) injury model. After CTX injection, skeletal muscle undergoes degeneration, followed by inflammatory cell infiltration, and regeneration of new myofibers marked by internal nuclei. Although regeneration continues until 28 days after injury, the majority of new myofibers are formed by day 14 (35, 36). After CTX injury, *Col6a2^–/–^* and WT muscle fibers underwent degeneration, with infiltration of the muscle with inflammatory cells by day 3 and regeneration by day 7. However, regeneration in *Col6a2^–/–^* muscle was delayed, with persistent inflammatory infiltrates, increased prevalence of small myofibers/myotubes, and increased endomysial spaces at 14 days after injury (Figure 7A).

Restructuring of the ECM after injury and deposition of a provisional matrix is necessary to maintain tissue integrity and support regeneration and is often perturbed in muscle disease (37). Thus, we set out to evaluate the ECM components after injury in our model. The endomysial spaces at day 14 after injury did not stain positive with the Sirius red stain and muscle hydroxyproline content was not markedly increased after CTX injury, suggesting that the endomysial spaces contain ECM components other than fibrillar collagens (Figure 7B and Supplemental Figure 7A). Fibronectin is a TGF-β–responsive gene and a prominent and abundant component of provisional matrices, necessary for tissue repair (38, 39). Periostin is also a TGF-β–responsive gene that actuates the profibrotic effects of TGF-β in dystrophic muscle (40). Immunostaining and Western blotting for fibronectin and periostin showed rapid and dynamic changes in their expression after CTX injury. In the WT muscle, rapid and temporary overexpression of these proteins peaked at day 7, while in *Col6a2^–/–^* muscle, their overexpression was delayed, prolonged, and dampened (Figure 7, C and D). These data highlight the differences in provisional ECM components after injury that may contribute to suboptimal regeneration in collagen VI–deficient muscle.

### Absence of collagen VI reduces active TGF-β release in response to muscle injury.

TGF-β activation from the ECM typically occurs in response to a variety of stimuli, such as mechanical force (41–43) and induced proteolytic activity (for example by plasmin, kallikreins, or matrix metalloproteinases) (29, 44). Tissue injury, including skeletal muscle injury, causes increased TGF-β activity (45, 46). Given the altered TGF-β activation, ECM changes, and concurrent impaired regenerative capacity in *Col6a2^–/–^* muscle, we next evaluated the dynamics of TGF-β activation in *Col6a2^–/–^* muscle at different time points after CTX injury. WT muscle had a large increase in TGF-β levels (>20-fold) within 24 hours of CTX injection, which gradually declined during the later phases of muscle regeneration (Figure 7D). Although baseline levels of TGF-β were higher in *Col6a2^–/–^* muscle, in the acute phases after CTX injury, TGF-β release was markedly dampened compared with that in WT controls (Figure 7D). The TGF-β release dynamics closely mirrored the pattern and dynamics of periostin and fibronectin expression in the ECM.

We also evaluated the dynamics of collagen VI expression after CTX injury, which showed increased collagen VI expression during muscle regeneration, coinciding with decreasing TGF-β levels (Supplemental Figure 7, B and C). These findings suggest that collagen VI regulates TGF-β bioavailability in the skeletal muscle ECM after injury as well, allowing for a robust but temporary increase in TGF-β and its responsive proteins in early phases of regeneration followed by a choreographed decline in later phases of muscle regeneration.

### Decreased Ltbp4–TGF-β binding does not alter the Col6a2^–/–^ mouse muscle phenotype.

TGF-β is predominantly secreted as part of a large latent complex that includes the active TGF-β dimer that is non-covalently bound to its latency-associated peptide (LAP) and a latent TGF-β–binding protein (LTBP). Four LTBPs are known (LTBP1–LTBP4), of which LTBP1, LTBP3, and LTBP4 bind TGF-β with varying efficiencies (29, 47); however, their absence typically results in reduced TGF-β activity, presumably due to lower efficiency of TGF-β secretion and activation. Ltbp4 is the most abundant Ltbp in mouse skeletal muscle (Supplemental Figure 8) and is a modifier of disease in mouse models of muscular dystrophy (48) and individuals with Duchenne muscular dystrophy (49). In addition, stabilization of the hinge region of Ltbp4 protein reduces TGF-β activation and mitigates the muscular dystrophy phenotype in some mouse models of muscular dystrophy (50, 51).

**Figure 8:**
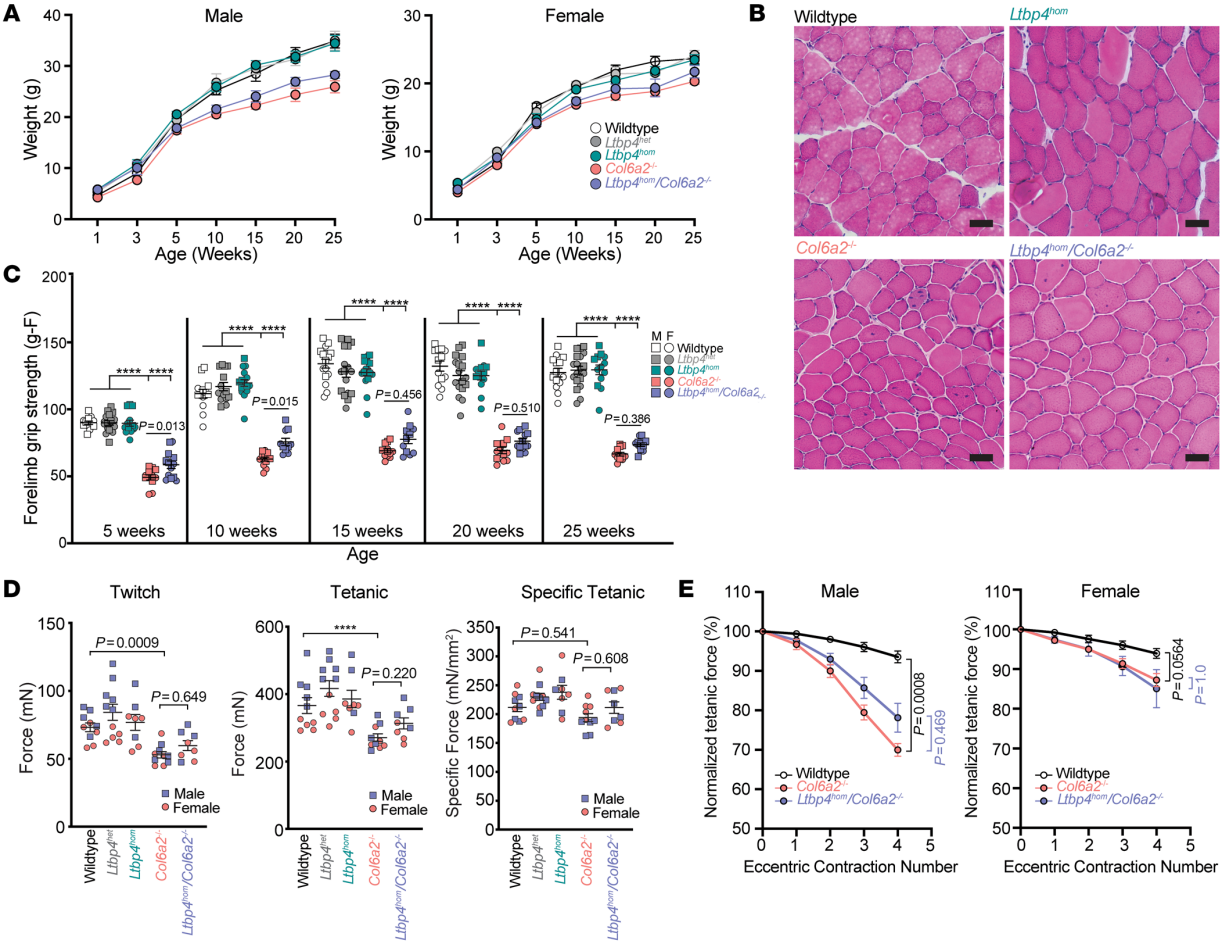
Decreased Ltbp4*–*TGF-β binding does not alter the *Col6a2^–/–^* mouse muscle phenotype. (**A**) Double-homozygous *Ltbp4^hom^*/*Col6a2^–/–^* mice have weight similar to that of *Col6a2^–/–^* mice and are smaller and lighter than WT and *Ltbp4^het^* and *Ltbp4^hom^* littermates. (**B**) H&E staining of tibialis anterior muscle of double-homozygous *Ltbp4^hom^/Col6a2^–/–^* mice shows dystrophic features similar to those in *Col6a2^–/–^* mice (25 weeks old). Scale bars: 50 μm. (**C**) Although *Ltbp4^hom^*/*Col6a2^–/–^* mice have slightly higher forelimb grip strength compared with *Col6a2^–/–^* mice, it remains markedly lower than that of WT *Ltbp4^het^* and *Ltbp4^hom^* littermates. (**D**) Physiological parameters in isolated EDL muscle of 25-week-old *Ltbp4^hom^*/*Col6a2^–/–^* mice were unchanged in comparison with *Col6a2^–/–^* mice. For **C** and **D**, statistical comparisons were performed by 2-way ANOVA and Tukey’s adjustment for multiple comparisons. ****P *<* 0.0001. (**E**) After repeated eccentric contractions, tetanic force declined precipitously in isolated double-homozygous *Ltbp4^hom^*/*Col6a2^–/–^* and *Col6a2^–/–^* EDL muscles, more prominently in male animals compared with female animals. Statistical analysis was performed using linear mixed models and Bonferroni adjustment for multiple comparisons. Error bars represent SEM.

Since our *Col6a2^–/–^* mice showed altered TGF-β activation in skeletal muscle with evidence of baseline overactivity of TGF-β pathways, we hypothesized that reducing Ltbp4–TGF-β interaction might improve the disease phenotype in our mouse model. We tested this hypothesis by crossing the *Col6a2^–/–^* mice to a knockin mutant *Ltbp4* mouse model (*Ltbp4^c/s^*) in which a critical cysteine residue in the third TGF-β–binding, 8-cysteine domain of Ltbp4 is mutated (52). In homozygous mice with this knockin mutation (*Ltbp4^hom^*), Ltbp4 does not associate with TGF-β–LAP but is normally secreted and localizes to the ECM (52). We first reconfirmed the normal localization of Ltbp4 in skeletal muscle in the *Ltbp4^het^* and *Ltbp4^hom^* transgenics and in double-homozygote *Ltbp4^hom^*/*Col6a2^–/–^* mice (Supplemental Figure 8B). We then measured their weight and grip strength and analyzed the muscle histology and ex vivo physiology parameters. Although some of these parameters trended slightly higher, these studies failed to detect a meaningful improvement in any of the functional, physiological, or histological assays (Figure 8). This suggests that modulating the Ltbp4–TGF-β interaction is insufficient to address the consequences of TGF-β dysregulation in the *Col6a2^–/–^* muscle ECM.

## Discussion

In this work, we report a mouse model of *COL6*-RD with homozygous deletion of the *Col6a2* genomic locus resulting in complete absence of collagen VI protein from the skeletal muscle ECM. Using standardized behavioral tests and physiological parameters, we establish a comprehensive natural history study of this preclinical model. *Col6a2^–/–^* mice have a normal lifespan but develop muscle atrophy and weakness with histological hallmarks of muscular dystrophy and fibrosis (Figures 1–4 and Supplemental Figures 1–4). Similar to individuals with heterozygous loss-of-function variants in collagen VI–related genes, heterozygous *Col6a2^+/–^* mice show no signs of muscle disease. Likewise, we did not detect phenotypic sexual dimorphism in male and female mice; the only exception was relative protection of female *Col6a2^–/–^* mice against eccentric contraction–induced muscle injury ex vivo (Figure 3F and Supplemental Figure 3B). Similar sex differences in this assay have been reported in female mdx mice, a mouse model of dystrophin-deficient muscular dystrophy, and attributed to estrogen availability (53). Thus, sex differences must be considered in designing future studies that use this physiological parameter as a primary readout. Another possibility for the observed sex differences in the eccentric contraction induced muscle injury assay could be related tothe relatively small sample size in muscle contractility studies (*n* = 5 male, 5 female), a limitation potentially confounding our findings. We did not find sex differences in the related in vivo downhill treadmill run assay in *Col6a2^–/–^* mice (Figure 3, G and H).

COL6-RDs are a progressive disease in humans; however, we did not note a similar progressive course in *Col6a2^–/–^* mice up to 60 weeks of age. While we did not notice a change in the lifespan of *Col6a2^–/–^* mice, we did not systematically assess the disease phenotype in mice older than 60 weeks of age, and thus, it remains possible that they develop more severe disease in extremes of age. Extramuscular phenotypic studies were not the focus of our current study, but trabecular bone loss has been reported in *Col6a2^–/–^* mice previously (54) and is a common clinical finding in the human disease. Our RNA-Seq study of mouse muscle tissue identified an upregulation of the TGF-β pathway–related genes in juvenile and adult *Col6a2^–/–^* muscle (Figure 5). Upregulation of the TGF-β pathway was also reported as the leading finding in transcriptomic studies of *COL6*-RD human muscle biopsies (28) and thus provides further validity to our mouse model and its relevance to the molecular mechanisms of *COL6*-RDs. Overall, *Col6a2^–/–^* mice manifest with a phenotype and molecular signature that recapitulate the human disease, albeit with less severity and without notable progression.

The reason for the observed difference between the human disease and mouse models is not clear, but it has been a consistent finding in other mouse models of *COL6*-RDs (Supplemental Table 1). While other collagen VI components are not able to compensate for the absence of the obligatory col6a2 chain in the structure of collagen VI, other candidate microfibrillar proteins can theoretically compensate for collagen VI function. For example, we noted an overexpression of fibrillin-1 (1.6-fold) in the 25-week-old mouse RNA-Seq dataset (Supplemental Data File 1). Fibrillin-1 is a microfibrillar ECM component with ultrastructural similarity to collagen VI and thus may partially compensate for its function and contribute to the observed reduced phenotypic severity.

Fibrosis is a nearly universal histological feature in muscular dystrophies. In *Col6a2^–/–^* mice, we noted a gradual increase in endomysial fibrosis over time (Figure 4, A and B, and Supplemental Figures 4 and 5). Ultrastructural studies also demonstrated morphological changes in the endomysial ECM and fibrillar collagen (predominantly collagen I and collagen III) organization (Figure 4C). Importantly, muscle atrophy and weakness at 5 weeks of age preceded the appearance of histologically overt fibrosis. This suggests that in early phases of the disease, the pathogenic processes distinct from muscle fibrosis result in muscle weakness in *Col6a2^–/–^* mice. We also assessed the passive biomechanical properties of muscle in *Col6a2^–/–^* muscle and found a marked increase in the series modulus of elasticity and damping (Figure 4, D and E). The histological counterpart of these physiological parameters is not precisely known and includes the intracellular myofibril-associated titin protein, the sarcolemma, and the myotendinous junction as well as the ECM (26); however, the concurrent perturbations in the series modulus of elasticity and damping are most consistent with altered ECM in *Col6a2^–/–^* muscle. Changes in passive mechanical properties of muscle can contribute to decreased force transmission efficiency (55), which may explain the discrepancy between the substantial decrease in maximal tetanic force and the lack of a corresponding decrease in specific force in isolated *Col6a2^–/–^* EDL muscle (Figure 3, C and D). Changes in passive mechanical properties of muscle also alter mechanical signal transduction and regulation of signaling proteins and growth factors such as TGF-β (56, 57). Thus, they may further contribute to the dysregulation of TGF-β and similar growth factors that maintain normal muscle function and its regenerative response to injury.

TGF-β overactivity in resting muscle was a prominent feature in *Col6a2^–/–^* mice. TGF-β pathway upregulation has been reported in other muscular dystrophies and linked to muscle fibrosis (58–60). In addition to its profibrotic effects, the TGF-β pathway contributes to several other downstream alterations in cellular function, including autophagy flux, mitochondrial abnormalities, and apoptosis (61), all of which have been previously reported in *Col6a1^–/–^* mice (21, 22). In the juvenile *Col6a2^–/–^* animals (5 weeks old) before the development of histological fibrosis and in *COL6*-RD patient muscle biopsies without overt fibrosis, we detected a dramatic upregulation of TGF-β pathway signaling (28). This is in contrast with similar studies in Duchenne muscular dystrophy that show increased TGF-β pathway signaling in later stages of the disease after an initial inflammatory phase (62). Thus, TGF-β overactivity in *Col6a2^–/–^* mice may cause muscle weakness and atrophy, independent of its profibrotic effects that manifest with fibrosis later in the disease course.

In addition to muscle weakness and fibrosis, *Col6a2^–/–^* mice show decreased regeneration capacity in response to CTX-induced muscle injury (Figure 7A). We found divergent effects on TGF-β availability in resting muscle and in acute phases of response to injury in *Col6a2^–/–^* muscle. While TGF-β was overactive at rest, we noted 2 distinct alterations in TGF-β bioavailability during muscle regeneration. First, there was a relative deficiency of active TGF-β in *Col6a2^–/–^* muscle in the early post-injury period (Figure 7D), which coincided with myoblast and fibroblast proliferation and immune cell infiltration of the injured muscle tissue. Relatively reduced TGF-β levels at this juncture may contribute to inadequate myoblast proliferation or alter provisional matrix deposition that is necessary for normal muscle regeneration. Second, there was a relative increase in active TGF-β in later phases of regeneration, which coincided with myotube-myofiber fusion. Increased TGF-β signaling has been shown to delay muscle regeneration by preventing myotube fusion (45, 63) and thus may further hamper the normal regenerative response in *Col6a2^–/–^* muscle and is consistent with the delayed regeneration noted in *Col6a2^–/–^* muscle (Figure 7A). Thus, depending on time points, both a relative deficiency and a relative overactivity of TGF-β signaling may contribute to the disease pathogenesis in *Col6a2^–/–^* muscle. Future studies are needed to evaluate the respective contributions of altered TGF-β activity to muscle regeneration at these different time points.

A counterintuitive finding was that while fibrosis was increased at steady state in association with TGF-β overactivity in *Col6a2^–/–^* muscle, it did not directly correlate with TGF-β levels in the early post-injury period (Figure 7D and Supplemental Figure 7A). We postulate that development of fibrosis likely requires chronic and sustained exposure to TGF-β and its downstream effectors. In other words, the duration of the summative exposure of skeletal muscle to TGF-β is an important variable that contributes to development of fibrosis via its downstream profibrotic effectors (e.g., periostin). The timing of emergence of fibrosis in the older animals despite a prominent increase in TGF-β signaling as early as 5 weeks of age illustrates the importance of this summative exposure for emergence of fibrosis, with potential impact on the timing of antifibrotic therapeutic interventions that aim to prevent it or mitigate its effects.

Our data suggest that collagen VI or its binding partners in the ECM play a direct role in regulating TGF-β activity in skeletal muscle. Specifically, using collagen VI co-IP/mass spectrometry experiments, we identified several TGF-β–binding or microfibril-associated proteins that bind collagen VI and are also known to regulate TGF-β activity in skeletal muscle, such as decorin, fibrillin-1, and Mfap2 (also known as Magp-1) (31, 64). Consistent with ECM regulation of TGF-β activity, increased TGF-β activity in *Col6a2^–/–^* muscle was not due to a change in its transcription or translation (Supplemental Figure 6). Instead, our co-IP and native gel data showed a change in TGF-β protein interactions (Figure 6, D and F), which likely underlies its overactivity in resting muscle. During muscle regeneration, we noted dynamic changes in TGF-β levels and provisional ECM composition. Notably, decrease in active TGF-β levels coincided with increasing collagen VI levels (Supplemental Figure 7, B and C) consistent with a functional and molecular interaction between collagen VI and active TGF-β in the ECM. We thus surmise that the collagen VI deficiency in the ECM interferes with the proper regulation of active TGF-β availability, which in turn results in a relative deficiency of TGF-β when it is in high demand and its overactivity when it is to be sequestered again.

We next set out to reduce TGF-β ECM availability by abrogating its binding to Ltbp4 and tested the effects on the *Col6a2^–/–^* mouse myopathic phenotype. The rationale for this experiment was based on the growing body of literature linking LTBP4 function and stability to muscular dystrophy phenotype (48–51). Crossing the *Col6a2^–/–^* mice with the *Ltbp4^c/s^* mice failed to improve muscle strength, histology, or physiological parameters (Figure 8). These negative data suggest that TGF-β overactivity in *Col6a2^–/–^* mice may occur downstream of Ltbp4–TGF-β binding or other TGF-β–binding Ltbps (Ltbp1 and Ltbp3) may compensate for abrogation of Ltbp4–TGF-β binding and thus dampen its potential therapeutic effects. Future interventional studies that more effectively sequester TGF-β in the ECM or specifically inhibit its receptors on myofibers are needed to pursue the efficacy of targeting this pathway as a therapeutic strategy for *COL6*-RDs.

In summary, our study establishes and validates a new mouse model of *COL6*-RD with direct phenotypic and molecular relevance to the human disease and proposes that the collagen VI–deficient matrix results in a primary and substantial dysregulation of dynamic TGF-β bioavailability as a specific contributor to the disease pathogenesis. This study paves the way for future mechanistic studies into the role of ECM in disease pathogenesis in muscular dystrophy as well as interventional studies that target the TGF-β pathway in *COL6*-RDs.

## Methods

### Sex as a biological variable.

Our study examined male and female animals, and similar findings are reported for both sexes in the vast majority of the assays. Sex-dimorphic effects are highlighted when they were encountered.

### Mouse studies and housing conditions.

The mouse strain C57BL/6N-*Col6a2*^tm1(KOMP)Vlcg/MbpMmucd^, RRID:MMRRC_047168-UCD, was obtained from the Mutant Mouse Resource and Research Center (MMRRC) at UCD, an NIH-funded strain repository, and was donated to the MMRRC by the Knockout Mouse Project (KOMP) Repository, UCD, originating from David Valenzuela and George Yancopoulos, Regeneron Pharmaceuticals Inc., and Kent Lloyd, UCD Mouse Biology Program.

After the *Col6a2*^tm1(KOMP)Vlcg^ mice were backcrossed 5 generations onto a C57BL/6J background, the colony was maintained at the intramural NIH Building 35 vivarium with a regular 12-hour light/12-hour dark cycle, room temperature at 20°C–25°C, and a relative humidity of 40%–65% with ad libitum access to untreated drinking water and chow. Heterozygous (*Col6a2^+/–^*) breeders were used to generate WT controls, *Col6a2^+/–^* controls/breeders, and homozygous *Col6a2*^tm1(KOMP)Vlcg^ (*Col6a2^–/–^*) transgenic animals.

The *Ltbp4^c/s^* knockin mice were generated by InGenious Targeting Laboratory as previously described (52) with an *Ltbp4* p.Cys1260Ser substitution and re-derived at the National Institute of Mental Health (NIMH) transgenic mouse core from mouse sperm straws provided by Daniel Rifkin’s laboratory at New York University.

*Col6a2* genotyping was performed using endpoint PCR and gel electrophoresis with the following primers: *Col6a2* WT: forward 5′-TGCCCTCTCTGTTCTTCATGTACCC-3′, reverse 5′-GACTTTGGTCTGAAAGGAACACCCG--3′; *Col6a2* knockout: forward 5′-ACTTGCTTTAAAAAACCTCCCACA-3′, reverse 5′-GCCTGGGGTAAGGCCCTATT-3′. The *Ltbp4^c/s^* genotyping was performed by PCR using *Ltbp4* forward primer 5′-GTCTACAGAGTGGGTTGCAGG-3′ and *Ltbp4* reverse primer 5′-GCACCACTAACCCAATCCTTAG-3′ followed by restriction digest with *BstAPI*, which only digested the WT allele, and gel electrophoresis.

### Functional and in vivo studies.

Animals were weighed before each test. The genotype of the mice was originally masked; however, because of the small size and apparent weakness of the homozygous animals, masking became ineffective. Functional measurements were performed on the same equipment, in the same room, and by the same operator in the same 2-hour window of the light cycle. For details of each functional test, see Supplemental Methods.

### Ex vivo force measurements.

Ex vivo force measurement was performed in accordance with TREAT-NMD standard operating procedure DMD_M.1.2.002 (https://www.treat-nmd.org/wp-content/uploads/2023/07/DMD_M.1.2.002.pdf). Physiological analysis was performed on isolated EDL muscles using a dual-mode servomotor apparatus and software (Aurora Scientific Inc.) as previously described (65). Briefly, after dissection, EDL muscle was submerged in a bath of carbogen-equilibrated (95% O_2_/5% CO_2_) Ringer’s solution at 30°C. Optimal length (L_o_) was experimentally determined and measured using calipers. Maximum tetanic force was measured after stimulation of 500-millisecond duration and of increasing frequency (100–200 Hz). The eccentric contraction stimulation paradigm included a 150-Hz, 700-millisecond pulse, where a stretch of 10% L_o_ was administered in the last 200 milliseconds of the contraction. Five contractions separated by 3 minutes of rest in between were administered, and the tetanic force prior to the stretch was compared with baseline. The physiological cross-sectional area was determined as previously described (65).

Passive mechanical properties of the EDL muscle were measured based on a viscoelastic A.V. Hill model, as previously described (25). Briefly, after dissection and experimental determination of L_o_, each muscle was subjected to a stretch of 5% L_o_ at a rate of about 50 L_o_/s, held for 7.0 seconds before return to L_o_. Series and parallel elastic elements and damping were then determined using an exponential fit as previously described (25).

### Histology and light microscopy.

Mouse muscles were dissected and frozen in cooled isopentane, and 8 μm cryosections were collected on glass slides and stored at –80°C. For hematoxylin and eosin stain, sections were incubated in Harris hematoxylin solution (VWR, catalog 95057-858) for 5 minutes, washed with tap water, and incubated with eosin (Sigma-Aldrich, catalog 318906) for 1 minute. The slides were then washed in dH_2_O, dehydrated in serially graded ethanol solutions, and cleared in xylene, before mounting in Permount (Fisher Scientific, catalog SP15-100) and coverslipping.

For the Sirius red stain, the sections were incubated in 95% ethanol for 2 minutes, counterstained with 0.5% Fast Green (Sigma-Aldrich, catalog 68724) in PBS (wt/vol) for 5 minutes, washed with dH_2_O, incubated with Sirius red solution (0.1% wt/vol Direct Red 80 [Sigma-Aldrich, catalog 365548] in picric acid) for 30 minutes, rinsed with 0.5% vol/vol acetic acid, dehydrated serially in graded ethanol solutions, cleared in xylene, mounted in Permount, and coverslipped.

The slides were imaged using a Nikon Eclipse Ti light microscope, and tiled images were obtained using a Nikon Digital DS-Fi1 camera.

### Immunofluorescence.

Eight-micrometer cryosections were collected on glass slides, fixed in 4% paraformaldehyde for 10–15 minutes at room temperature, and washed in PBS. Sections were blocked with 5% normal goat serum (NGS) or 10% fetal bovine serum (FBS) and 0.5% Triton for 1 hour at room temperature before incubation with primary antibodies overnight at 4°C. After washing with PBS, the sections were incubated with diluted secondary antibodies for 1 hour at room temperature, washed with PBS, and incubated with DAPI solution before mounting with Flouromount-G (Invitrogen, catalog 00-4958-02) and coverslipping. For wheat germ agglutinin (WGA) staining, the sections were incubated in 5 μg/mL Alexa Fluor 488– or 633–conjugated WGA in PBS for 10 minutes before mounting. The sections were imaged using a Nikon Eclipse Ti epifluorescence microscope and a Nikon Digital DS-Fi1 camera. Confocal microscopy was performed on a Leica TCS SP5 II confocal microscope. The primary antibodies and dilutions were as follows: dystrophin, Sigma-Aldrich D8403 (MANDRA1), mouse IgG1 (1:100 in 5% NGS); collagen VI, Abcam ab6588, rabbit (1:200 in 5% NGS); p-SMAD3, Abcam ab52903, rabbit (1:100 in 10% FBS); LTBP4, R&D Systems AF2885, goat (1:100 in 5% NGS); myosin heavy chain I (MyoHC I), Developmental Studies Hybridoma Bank (DSHB; Iowa City, Iowa, USA) BA-D5, mouse IgG2b (1:20 in 5% NGS); MyoHC IIa, DSHB SC-71, mouse IgG1 (1:20 in 5% NGS); MyoHC IIb, DSHB BF-F3, mouse IgM (1:20 in 5% NGS); periostin, Novus NBP1-30042, rabbit (1:100 in 10% FBS); fibronectin, Sigma-Aldrich F3648, rabbit (1:200 in 10% FBS).

### Quantification of histological parameters and morphometry.

We quantified the fibrotic area of the muscle using the Sirius red stain images by splitting the RGB channels, thresholding the green channel, highlighting the extracellular fibrillar collagen content, and measuring the percentage area limited to threshold in Fiji/ImageJ (http://imagej.nih.gov/ij/). Muscle morphometry was performed using the Myosoft Fiji/ImageJ plugin (24). Internalized nuclei were manually tallied using the cell counter tool in Fiji/ImageJ.

### Muscle ECM scanning electron microscopy.

The muscle tissue was dissected from euthanized animals, fixed in 4% formaldehyde, and processed as described previously (66, 67). Briefly, disc-shaped, 2-mm-thick pieces of muscle were fixed in Karnovsky’s fixative (4% glutaraldehyde, 4% paraformaldehyde, 0.2 mol/L sodium cacodylate buffer, pH 7.4) overnight, washed in 0.1 mol/L sodium cacodylate buffer, and decellularized in 10% NaOH at room temperature for 4–7 days. The decellularized samples were then prepared for scanning electron microscopy in 1% aqueous tannic acid, and postfixed in 1% aqueous osmium tetroxide overnight. Samples were then dehydrated in ethanol, critical-point-dried in liquid CO_2_, mounted on aluminum stubs with carbon tape, sputter coated, and photographed with a Thermo Apreo Volume Scope scanning electron microscope at an accelerating voltage of 2 kV.

### Muscle fibroblast isolation and conditioned medium collection.

Skeletal muscle fibroblasts were isolated by adaptation of a previously described protocol (68). Quadriceps muscle from 3-week-old animals was dissected, minced using sterile razor blades, and digested in a 5 mL tube containing 3–5 mL of the digestion mix at 37°C for 90 minutes: collagenase type V (Sigma-Aldrich, C9263; 5 mg/mL) and dispase II (Gibco, 17105041; 3.5 mg/mL) in DPBS (Gibco, 14190094). After the digestion, an equal volume of growth medium (IMDM with Glutamax, Gibco, 31980022; 20% FBS; 1% chick embryo extract; and 1% penicillin/streptomycin) was added to the digestion mix. The neutralized digestion mix was then filtered and spun down to isolate the cells, which were plated in a 10 cm culture dish. After 30 minutes, non-adherent cells were removed, and the adherent cells were used for further studies. Only cells with fewer than 5 passage numbers were used. The adherent fibroblasts were plated at about 7,500 cells/cm^2^ in fibroblast growth medium (DMEM/F12, 10% FBS). After 7–10 days, the cells were washed with PBS, F10 medium without serum was added (62.5 μL/cm^2^ growth area) and collected after 16 hours and spun down at 300*g* for 5 minutes, and supernatant (conditioned medium) was stored at –80°C.

### TGF-β activity detection assay.

The HEK293 luciferase reporter cell line (HEK293-Luc) was a gift from Tejvir Khurana (University of Pennsylvania, Philadelphia, Pennsylvania, USA). HEK293 cells were stably transduced with a transgene with a promoter including a SMAD-binding element (CAGA)_12_ upstream of firefly luciferase transgene. The HEK293-Luc cells were plated at 30,000 cells/cm^2^ in 24-well plates in growth medium (DMEM, 10% FBS). After 24 hours, the cells were gently washed with PBS twice. Serum-free F10 medium with or without recombinant TGF-β1 (PeproTech, 100-21), conditioned medium from WT or *Col6a2^–/–^* fibroblast cultures, and/or neutralizing TGF-β antibody (clone 1D11, Invitrogen, MA5-23795) was added. After 16 hours, the cells were lysed with a Bright Glo Luciferase assay system (Promega, E2610) per the manufacturer’s recommendations, and luminescence was measured using a plate reader.

### Protein extraction, gel electrophoresis, and immunoblotting.

The muscle tissue was minced and mechanically disrupted using a glass tissue grinder and lysed in approximately 1:10 (wt/vol, mg of tissue/μL) of NP-40 lysis buffer (20 mM Tris-HCl [pH 7.4], 150 mM NaCl, 100 mM EDTA [pH 7.4], 1% NP-40; Calbiochem, catalog 492016) with protease inhibitors (cOmplete, Mini, EDTA-free Protease Inhibitor Cocktail, Sigma-Aldrich, catalog 11836170001) and phosphatase inhibitors (Phostop, Roche, catalog 4906845001). We then centrifuged the extracts at 14,000*g* at 4°C for 15 minutes and collected the supernatant and determined the protein concentration using the BCA protein assay kit (Pierce, catalog 23225). Thirty micrograms of total protein was reduced and denatured by incubation in LDS sample buffer (Invitrogen, NP0007) and 10 mM DTT at 95°C for 5 minutes, separated on a NuPAGE 4%–12% Bis-Tris gel (Invitrogen catalog NP0322BOX) in MOPS buffer, and transferred to a PVDF membrane. The membranes were washed, blocked in Intercept blocking buffer (LI-COR, catalog 927-70001) or 5% milk in TBS-T (10 mM Tris-HCl [pH 7.4], 150 mM NaCl, 0.05% Tween) for 1 hour at room temperature, and incubated with the primary antibodies overnight at 4°C. Bound antibodies were detected with the species-appropriate secondary antibodies by a ChemiDoc XRS (Bio-Rad) imager after enhanced chemiluminescence (Supersignal West Pico Plus, Thermo Fisher Scientific, catalog 34577) or a LI-COR Odyssey DLx scanner for infrared fluorescent-tagged secondary antibodies. Densitometry of the bands was performed with Fiji/ImageJ.

Antibodies and their dilutions used in Western blotting were as follows: collagen VI, Abcam ab6588, rabbit (1:1,000 in 5% BSA); p-SMAD3, Epitomics 1880-1, rabbit (1:500 in 5% BSA); SMAD3, Cell Signaling 9513, rabbit (1:500 in 5% BSA); TGF-β1, R&D Systems MAB240, mouse (1:500 in 5% BSA); GAPDH, EMD Millipore MAB374, mouse (1:5,000 in 5% milk); fibronectin, Sigma-Aldrich F3648, rabbit (1:2,500 in Intercept); periostin, Novus NBP1-30042, rabbit (1:1,000 in 5% milk).

### Native gel electrophoresis.

Protein samples were prepared using the NativePAGE sample preparation kit (Thermo Fisher Scientific, BN2008) in 1% digitonin or 1% NP-40. Composite 0.5% agarose/2.5% polyacrylamide gels were cast as previously described (69). The gels were run based on NativePAGE system instructions (Invitrogen) with a few modifications. Tris-borate (TBE; 0.45 M, pH 8.0) buffer was used in the lower chamber. After addition of dark cathode buffer in TBE in the upper chamber, the gel was run at 150 V for 15 minutes on ice. Then, the upper chamber buffer was switched to light cathode buffer in TBE, the gel was run for an additional 50 minutes at 150 V on ice, and the proteins were then transferred to a PVDF membrane, which was incubated with 8% acetic acid after transfer for 15 minutes and washed in dH_2_O. The membrane was washed with methanol before immunoblotting.

### Cardiotoxin injury.

Mice (~15 weeks of age) were anesthetized with 2%–3% isoflurane. A sterile solution of cardiotoxin (CTX; Sigma-Aldrich, 217503) in sterile PBS was injected intramuscularly (~100 μL of 10 μg/mL solution) into the tibialis anterior muscle. The contralateral muscle was injected with approximately 100 μL of sterile PBS and used as a negative control, and the muscles were collected at different time points.

Additional methods are given in Supplemental Methods.

### Statistics.

Two-way analysis of variance (ANOVA) with Tukey’s adjustment for multiple comparisons was used for statistical comparisons, with sex (male or female) and genotype (WT, *Col6a2^+/–^*, or *Col6a2^–/–^*) as categorical independent variables. When stratified by sex, ordinary 1-way ANOVA with Tukey’s adjustment for multiple comparisons was used for comparisons. Hanging wire test data were analyzed using time-to-event (defined as falling) analysis on a Cox proportional hazard model with Bonferroni-adjusted pairwise comparisons across the genotypes. Eccentric contraction data were analyzed using linear mixed models, with score as the outcome variable and group as the main covariate. Repeated observations were controlled for within subject. Overall group effect was described using *F* statistics, and pairwise comparisons between groups. Downhill run data were analyzed using 1-way ANOVA. A separate ANOVA was conducted at baseline and after downhill run. Western blot protein quantification data and hydroxyproline assays were compared using a parametric, 2-tailed unpaired *t* test with Welch’s correction. For the RNA-Seq data, statistical comparisons were performed using analysis of covariance under Akaike information criterion–step (AIC-step) optimization and Benjamini-Hochberg false discovery rate multiple-comparison correction condition, correcting for sex after normalization and noise filtering. Power calculations for the *Col6a2^–/–^*/*Ltbp4^c/s^* double-homozygous mice were based on our desired detection of biological effect of 25% or more on grip strength being deemed as meaningful, and determined that we could arrive at conclusive studies (0.90 power) by testing 6 animals in each group. Outliers were identified by the ROUT method (GraphPad Prism v10.10) with a *Q* of 0.5%.

### Study approvals.

All animal studies were performed in accordance with NIH or Johns Hopkins University IACUC–approved protocols (NIH: ASP-1337 and ASP-1469; JHU: MO22M295).

### Data availability.

All data generated or analyzed are published as part of this article or its supplemental material, including the Supporting Data Values file. The transcriptomic datasets generated and analyzed during this study are available in the NCBI’s Gene Expression Omnibus repository (https://www.ncbi.nlm.nih.gov/geo; GSE228223). Requests for raw and analyzed data and materials related to this article but not included in it or in its supplemental material will be reviewed by the respective institution to verify whether the request is subject to any intellectual property or confidentiality obligations. Any data and materials that can be shared will be released via a material transfer agreement.

## Author contributions

PM designed and performed laboratory experiments, analyzed data, and drafted the manuscript. JR, YZ, MAN, PY, HH, TO, and DAS performed laboratory experiments and analyzed data. KJ, GN, TO, SS, TJR, and DBR analyzed data. CGB designed and oversaw the study, analyzed data, and revised the manuscript. All authors reviewed and edited the manuscript.

## Supplementary Material

Supplemental data

Supplemental data set 1

Supplemental data set 2

Unedited blot and gel images

Supporting data values

## Version 1 05/01/2025

Electronic publication

## References

[1] Bonnemann CG (2011). The collagen VI-related myopathies: muscle meets its matrix. Nat Rev Neurol.

[3] Mohassel P (2018). Extracellular matrix-driven congenital muscular dystrophies. Matrix Biol.

[4] Behan WM (2003). Muscle fibrillin deficiency in Marfan’s syndrome myopathy. J Neurol Neurosurg Psychiatry.

[5] Veilleux LN (2017). Muscle abnormalities in osteogenesis imperfecta. J Musculoskelet Neuronal Interact.

[6] Mohassel P (2019). Dominant collagen XII mutations cause a distal myopathy. Ann Clin Transl Neurol.

[7] Kielty CM (1991). Isolation and ultrastructural analysis of microfibrillar structures from foetal bovine elastic tissues. Relative abundance and supramolecular architecture of type VI collagen assemblies and fibrillin. J Cell Sci.

[8] Godwin ARF (2017). Defining the hierarchical organisation of collagen VI microfibrils at nanometre to micrometre length scales. Acta Biomater.

[9] Gara SK (2011). Differential and restricted expression of novel collagen VI chains in mouse. Matrix Biol.

[10] Gara SK (2008). Three novel collagen VI chains with high homology to the alpha3 chain. J Biol Chem.

[11] Lamande SR, Bateman JF (2018). Collagen VI disorders: insights on form and function in the extracellular matrix and beyond. Matrix Biol.

[13] Dowling JJ (2021). Molecular and cellular basis of genetically inherited skeletal muscle disorders. Nat Rev Mol Cell Biol.

[14] Pepe G (2002). Bethlem myopathy (BETHLEM) and Ullrich scleroatonic muscular dystrophy: 100th ENMC international workshop, 23-24 November 2001, Naarden, The Netherlands. Neuromuscul Disord.

[15] Zou Y (2008). Muscle interstitial fibroblasts are the main source of collagen VI synthesis in skeletal muscle: implications for congenital muscular dystrophy types Ullrich and Bethlem. J Neuropathol Exp Neurol.

[16] Bonaldo P (1998). Collagen VI deficiency induces early onset myopathy in the mouse: an animal model for Bethlem myopathy. Hum Mol Genet.

[17] Noguchi S (2016). Muscle weakness and fibrosis due to cell autonomous and non-cell autonomous events in collagen VI deficient congenital muscular dystrophy. EBioMedicine.

[18] Pan TC (2013). COL6A3 protein deficiency in mice leads to muscle and tendon defects similar to human collagen VI congenital muscular dystrophy. J Biol Chem.

[19] Pan TC (2014). A mouse model for dominant collagen VI disorders: heterozygous deletion of Col6a3 exon 16. J Biol Chem.

[20] de Greef JC (2016). Collagen VI deficiency reduces muscle pathology, but does not improve muscle function, in the γ-sarcoglycan-null mouse. Hum Mol Genet.

[21] Grumati P (2010). Autophagy is defective in collagen VI muscular dystrophies, and its reactivation rescues myofiber degeneration. Nat Med.

[22] Merlini L (2008). Cyclosporin A corrects mitochondrial dysfunction and muscle apoptosis in patients with collagen VI myopathies. Proc Natl Acad Sci U S A.

[23] Urciuolo A (2013). Collagen VI regulates satellite cell self-renewal and muscle regeneration. Nat Commun.

[24] Encarnacion-Rivera L (2020). Myosoft: an automated muscle histology analysis tool using machine learning algorithm utilizing FIJI/ImageJ software. PLoS One.

[25] Wolff AV (2006). Passive mechanical properties of maturing extensor digitorum longus are not affected by lack of dystrophin. Muscle Nerve.

[26] Binder-Markey BI (2021). Systematic review of skeletal muscle passive mechanics experimental methodology. J Biomech.

[27] Mariot V (2017). Downregulation of myostatin pathway in neuromuscular diseases may explain challenges of anti-myostatin therapeutic approaches. Nat Commun.

[28] Guadagnin E (2021). Transcriptome analysis of collagen VI-related muscular dystrophy muscle biopsies. Ann Clin Transl Neurol.

[29] Robertson IB, Rifkin DB (2016). Regulation of the bioavailability of TGF-β and TGF-β-related proteins. Cold Spring Harb Perspect Biol.

[30] Robertson IB (2017). The N-terminal region of fibrillin-1 mediates a bipartite interaction with LTBP1. Structure.

[31] Massam-Wu T (2010). Assembly of fibrillin microfibrils governs extracellular deposition of latent TGF beta. J Cell Sci.

[32] Isogai Z (2003). Latent transforming growth factor beta-binding protein 1 interacts with fibrillin and is a microfibril-associated protein. J Biol Chem.

[33] Wiberg C (2001). Biglycan and decorin bind close to the n-terminal region of the collagen VI triple helix. J Biol Chem.

[34] Finnis ML, Gibson MA (1997). Microfibril-associated glycoprotein-1 (MAGP-1) binds to the pepsin-resistant domain of the alpha3(VI) chain of type VI collagen. J Biol Chem.

[35] Couteaux R (1988). Regeneration of muscles after cardiotoxin injury. I. Cytological aspects. Biol Cell.

[36] Hardy D (2016). Comparative study of injury models for studying muscle regeneration in mice. PLoS One.

[37] Long AM (2023). Extracellular matrix contribution to disease progression and dysfunction in myopathy. Am J Physiol Cell Physiol.

[38] Zollinger AJ, Smith ML (2017). Fibronectin, the extracellular glue. Matrix Biol.

[39] Barker TH, Engler AJ (2017). The provisional matrix: setting the stage for tissue repair outcomes. Matrix Biol.

[40] Lorts A (2012). Deletion of periostin reduces muscular dystrophy and fibrosis in mice by modulating the transforming growth factor-β pathway. Proc Natl Acad Sci U S A.

[41] Buscemi L (2011). The single-molecule mechanics of the latent TGF-β1 complex. Curr Biol.

[42] Wipff PJ, Hinz B (2008). Integrins and the activation of latent transforming growth factor beta1 — an intimate relationship. Eur J Cell Biol.

[43] Shi M (2011). Latent TGF-β structure and activation. Nature.

[44] Lyons RM (1990). Mechanism of activation of latent recombinant transforming growth factor beta 1 by plasmin. J Cell Biol.

[45] Girardi F (2021). TGFβ signaling curbs cell fusion and muscle regeneration. Nat Commun.

[46] Kim J, Lee J (2017). Role of transforming growth factor-β in muscle damage and regeneration: focused on eccentric muscle contraction. J Exerc Rehabil.

[47] Rifkin DB (2018). LTBPs in biology and medicine: LTBP diseases. Matrix Biol.

[48] Heydemann A (2009). Latent TGF-beta-binding protein 4 modifies muscular dystrophy in mice. J Clin Invest.

[49] Flanigan KM (2013). LTBP4 genotype predicts age of ambulatory loss in Duchenne muscular dystrophy. Ann Neurol.

[50] Demonbreun AR (2021). Anti-latent TGFβ binding protein 4 antibody improves muscle function and reduces muscle fibrosis in muscular dystrophy. Sci Transl Med.

[51] Ceco E (2014). Targeting latent TGFβ release in muscular dystrophy. Sci Transl Med.

[52] Dabovic B (2015). Function of latent TGFβ binding protein 4 and fibulin 5 in elastogenesis and lung development. J Cell Physiol.

[53] Vang P (2021). Impact of estrogen deficiency on diaphragm and leg muscle contractile function in female mdx mice. PLoS One.

[54] Pham HT (2020). Collagen VIα2 chain deficiency causes trabecular bone loss by potentially promoting osteoclast differentiation through enhanced TNFα signaling. Sci Rep.

[55] Lieber RL, Binder-Markey BI (2021). Biochemical and structural basis of the passive mechanical properties of whole skeletal muscle. J Physiol.

[56] Munger JS (1999). The integrin alpha v beta 6 binds and activates latent TGF beta 1: a mechanism for regulating pulmonary inflammation and fibrosis. Cell.

[57] Yang Z (2007). Absence of integrin-mediated TGFbeta1 activation in vivo recapitulates the phenotype of TGFbeta1-null mice. J Cell Biol.

[58] Ceco E, McNally EM (2013). Modifying muscular dystrophy through transforming growth factor-β. FEBS J.

[59] Bernasconi P (1995). Expression of transforming growth factor-beta 1 in dystrophic patient muscles correlates with fibrosis. Pathogenetic role of a fibrogenic cytokine. J Clin Invest.

[60] Smith LR, Barton ER (2018). Regulation of fibrosis in muscular dystrophy. Matrix Biol.

[61] Zhang Y (2017). TGF-β family signaling in the control of cell proliferation and survival. Cold Spring Harb Perspect Biol.

[62] Chen YW (2005). Early onset of inflammation and later involvement of TGFbeta in Duchenne muscular dystrophy. Neurology.

[63] Melendez J (2021). TGFβ signalling acts as a molecular brake of myoblast fusion. Nat Commun.

[64] Broekelmann TJ (2020). Identification of the growth factor-binding sequence in the extracellular matrix protein MAGP-1. J Biol Chem.

[65] Zou Y (2014). Recessive and dominant mutations in COL12A1 cause a novel EDS/myopathy overlap syndrome in humans and mice. Hum Mol Genet.

[66] Ohtani O (1988). Collagen fibrillar networks as skeletal frameworks: a demonstration by cell-maceration/scanning electron microscope method. Arch Histol Cytol.

[67] Sleboda DA (2020). Diversity of extracellular matrix morphology in vertebrate skeletal muscle. J Morphol.

[68] Pimentel MR (2017). In vitro differentiation of mature myofibers for live imaging. J Vis Exp.

[69] Suh MH (2005). An agarose-acrylamide composite native gel system suitable for separating ultra-large protein complexes. Anal Biochem.

