# Supplementary material for: Collagen type VI regulates TGF-β bioavailability in skeletal muscle in mice
Source: J Clin Invest. 2025 May 1;135(9):e173354. doi: 10.1172/JCI173354 (PMC12043086; doi:10.1172/JCI173354)
Supplement: Supplemental data [file jci-135-173354-s268.pdf]

Supplementary table 1. Published mouse models of collagen VI-related dystrophy

Mouse line[Ref]	*Col6a1* KO[1]	*Col6a3* KO[2]	*Col6a1* KO[3]	*Col6a3* E16del[4]	*Col6a2* E5del[5]
Variant	*Col6a1* exon 2 deletion (out-of-frame)	*Col6a3* exon 15 and 16 deletion (out-of-frame)	*Col6a1* exon 10 deletion (out-of-frame)	*Col6a3* exon 16 deletion (in-frame)	*Col6a2* exon 5 deletion (in-frame)
Inheritance	Recessive	Recessive	Recessive	Dominant	Dominant
Lifespan	Normal	Normal	Normal	Normal	Normal
Grip weakness	Y	NA[a]	Y	Y	Y
Isolated muscle physiology defect	Y	Y	Y	Y	N
Fibrosis	Y	Y	Y	NA	N
Regeneration defect	Y	NA	Y	NA	NA

a NA= not assessed

References

1. Bonaldo P, Braghetta P, Zanetti M, Piccolo S, Volpin D, Bressan GM. Collagen VI deficiency induces early onset myopathy in the mouse: an animal model for Bethlem myopathy. Hum Mol Genet 1998;7:2135-2140.
2. Pan TC, Zhang RZ, Markova D, et al. COL6A3 protein deficiency in mice leads to muscle and tendon defects similar to human collagen VI congenital muscular dystrophy. J Biol Chem 2013;288:14320-14331.
3. Noguchi S, Ogawa M, Malicdan MC, Nonaka I, Nishino I. Muscle Weakness and Fibrosis Due to Cell Autonomous and Non-cell Autonomous Events in Collagen VI Deficient Congenital Muscular Dystrophy. EBioMedicine 2017;15:193-202.
4. Pan TC, Zhang RZ, Arita M, et al. A mouse model for dominant collagen VI disorders: heterozygous deletion of Col6a3 Exon 16. J Biol Chem 2014;289:10293-10307.
5. de Greef JC, Hamlyn R, Jensen BS, et al. Collagen VI deficiency reduces muscle pathology, but does not improve muscle function, in the gamma-sarcoglycan-null mouse. Hum Mol Genet 2016;25:1357-1369.
6. Noguchi S, Ogawa M, Malicdan MC, Nonaka I, Nishino I. Muscle Weakness and Fibrosis Due to Cell Autonomous and Non-cell Autonomous Events in Collagen VI Deficient Congenital Muscular Dystrophy. EBioMedicine 2016.
7. Grumati P, Coletto L, Sabatelli P, et al. Autophagy is defective in collagen VI muscular dystrophies, and its reactivation rescues myofiber degeneration. Nat Med 2010;16:1313-1320.
8. Merlini L, Angelin A, Tiepolo T, et al. Cyclosporin A corrects mitochondrial dysfunction and muscle apoptosis in patients with collagen VI myopathies. Proc Natl Acad Sci U S A 2008;105:5225-5229.
9. Urciuolo A, Quarta M, Morbidoni V, et al. Collagen VI regulates satellite cell self-renewal and muscle regeneration. Nat Commun 2013;4:1964.

Supplemental Methods

Grip strength

Forelimb grip strength was assessed using a grip strength meter with a mouse grid attachment (Bio-GS3, Bioseb) and in accordance with TREAT-NMD standard operating procedure MDC1A_M.2.2.001 Ver 1. At each timepoint, six peak force measurements were obtained for each mouse, with at least 1 minute of break in between trials, and averaged.

Hanging wire test

Hanging wire test was performed in accordance with TREAT-NMD standard operating procedure DMD_M.2.1.004. Animals were suspended by their forelimbs on a horizontal ~1.5 mm thick, 55 cm long metallic wire secured by two vertical posts, 35 cm above padded ground and the time when they fell was recorded. The test was stopped if the animal had not fallen after 600 s of suspension. Each animal was tested twice and their best performance was recorded.

Rotarod

Rotarod was performed after acclimation of the animals to the equipment (Ugo Basile, Cat# 47600) for a few minutes at low-speed (4-8 RPM) running. For the test, the rotation speed was increased from 2 to 40 RPM over a period of 2 minutes and the time when each animal fell from the apparatus was recorded.

Treadmill running, and downhill running paradigm

Treadmill running was performed after acclimation of the animals to the treadmill equipment (Exer 3/6, Columbus Instruments) and running protocol over 2-3 days. For the treadmill running test (at 0°of incline), the animals were run at a speed of 8 m/min for 2 minutes as warmup. The speed was increased by 1 m/min to a maximum of 12 m/min and maintained there until exhaustion. Exhaustion was defined as falling 10 times onto the shocking apparatus. The total distance was recorded. For the downhill running exercise, after warmup, the mice were run at 15° below horizontal for 30 minutes at 15 m/min. The downhill running was repeated daily over 3 consecutive days. The mice were intraperitoneally injected with Evan's blue dye (EBD-MP biomedicals, LLC Cat# 151108) at 50 mg/kg body weight prior to running downhill on day 3 and euthanized and dissected 7-8 hours later.

Activity meter

A force plate activity monitor (ActiVmeter, Bioseb) with infrared beam detection of rearing was used. The force plate was calibrated before each use and the animal was introduced to the activity cage and monitored for 1 hour, per manufacturer's specifications. Distance traveled, average speed, and rearing time were recorded every 5 minutes and analyzed using the manufacturer's software.

Voluntary wheel running

Mice were individually housed in cages equipped with a metered running wheel connected to a computer (Scurry Mouse Activity Monitoring wheel, Lafayette

Instruments Inc, Model 80820S). Digitized signals were processed by manufacturer's software (AWM software) and running distance at regular intervals were analyzed. The mice were acclimated to the apparatus for 24 hours and data analysis was performed on day 2 and 3.

TGFβ detection by enzyme-linked immunosorbent assay (ELISA)

Total TGFβ1 levels were quantified using the PicoKine TGFβ1 ELISA kit (Boster, cat# EK0515), following the manufacturer's recommendations. ELISA plates were incubated with sample lysates (5 µg of protein in 100 µl of diluent buffer) or a range of known recombinant TGFβ1 standards in duplicate and incubated at 37 °C for 90 minutes. After discarding the excess mix, biotinylated TGFβ1 antibody was incubated in each well for 60 minutes at 37 °C. Each well was washed three times and incubated with avidin-biotin-peroxidase complex at 37 °C for 30 minutes. Each well was washed 5 times and incubated with color developing reagent for 30 minutes at room temperature, neutralized with stop solution. Absorbance was measured using a plate reader at 450 nm and the values were modeled using a four-parameter logistic curve-fit, to calculate TGFβ1 concentrations.

Hydroxyproline assay

Hydroxyproline Assay was performed using the Hydroxproline Kit (Thermo Fisher MAK463). In brief, 10 mg of frozen muscle tissue was minced finely on dry ice and hydrolyzed with 100 µL NaOH and 100 µL H_2O at 100 degees C for 90 minutes, neutralized with 100 µL of 10 N HCl and centrifuged for 5 minutes at 14,000g at RT. 20

µL of each sample and standard was added to 96-well plate, and incubated with 90 µL of Oxidation/Reagent A buffer mix at RT for 10 minutes, mixed well with 90 uL of Reagent B and incubated at 37 degrees C for 90 minutes. The absorbance was then read at 560 nm with a plate reader.

Co-immunoprecipitation and data independent acquisition (DIA) mass spectrometry

Co-immunoprecipitation was performed using the Pierce magnetic bead co-IP kit (Thermo Fisher cat#: 88804). Mouse skeletal muscle lysates (1000 µg) were mixed with 1 µg of collagen VI (Ab6588) or TGFβ (clone 1D11) antibody in 500 µL of lysis buffer overnight at 4 degrees. The following day, 25 µL of Protein A/G magnetic beads were washed and incubatd with the antigen/antibody mix for 1 hour at room temperature. The beads were then washed with lysis buffer and ultra-pure water. Beads were then washed 3x in 10 mM triethylammonium bicarbonate (TEAB) buffer and suspended in a 40 µL of 10 mM TEAB, reduced with dithiothreitol (5.6 mM final concentration), alkylated with iodoacetamide (10 mM final concentration), and proteins were extracted with SP3 paramagnetic beads (GE Healthcare). Proteins were digested on-bead overnight at 37˚C with 1 µg trypsin/LysC mix (Thermo Pierce, cat# 40007). The digest supernatant was removed from beads, dried by vacuum centrifugation, and reconstituted in 100 µL 0.1% aqueous trifluoroacetic acid. Peptides were desalted using Oasis HLB uElution solid phase extraction plates (Waters) and eluates were dried. Desalted peptides were resuspended in 30 µl 2% acetonitrile in 0.1% formic acid and 6 µL was analyzed by nLC (Thermo Fisher Vanquish NEO) interfaced with HRMS (Thermo Fisher Orbitrap Exploris

480) as previously described[6]. Peptides were separated over 120 minutes using a linear gradient composed of mobile phases A (0.1% formic acid in water) and B (0.1% formic acid in 95% acetonitrile) at a flow rate of 0.3 µL/min, using the following gradient: initial conditions, 2%B; 2%B to 20%B in 50 minutes; 20%B to 30%B in 40 minutes; 30%B to 40%B in 15 minutes; 40%B to 100%B in 3 minutes; hold at 100%B for 5 minutes; 100%B to 2%B in 1 minute; column was equilibrated at 2%B over the final 6 minutes of the method. Spray voltage was set to 2kV and an RF lens setting of 50% was used. MS survey scans over the range 300-1250 m/z were collected at 120k resolution and following HCD fragmentation of precursor ions at 30% normalized collision energy, OW-DIA MS/MS scans were collected at 30k resolution in overlapping window mode, using 12 m/z-wide windows with 6 m/z overlaps. Raw files were demultiplexed with MSConvert[7, 8]. Data was searched within the Fragpipe interface, using MSFragger for library-free search[9], followed by Percolator for PSM validation[10], protein inference with ProteinProphet[11], and 1% PSM, peptide, and protein FDR filtering with Philosopher[12]. A spectral library was built with EasyPQP and DIA-NN was used for protein quantification[13]. The identified proteins were filtered based on p-value (<0.05).

RNA extraction and RNA-Seq

Sixty 10 µm cryosections of quadriceps muscle snap frozen in liquid nitrogen were collected in QIAzole (Qiagen Cat#79306). Four male and four female wildtype and *Col6a2*$^{-/-}$ mice were included at two different age groups (5 weeks and 25 weeks old). The RNA was isolated and purified as previously described[14]. RNA quality was

analyzed using a 2100 Bioanalyzer (Agilent Technologies, Santa Clara, CA). Samples that had a RIN/RINe ≥ 8 were used for RNA-Seq.

cDNA libraries were prepared according to Illumina protocols TruSeq RNA Sample Prep Kit starting with150 ng of total RNA. The cDNA was fragmented using a Covaris E210 and amplified using 12-15 cycles. Paired-end sequencing of the libraries was performed using the Illumina MiSeq platform with a target depth of 100 million reads of 150-base read pairs per library. Sequence output was processed via RTA and deplexed using CASAVA (http://www.illumina.com/software). Quality inspection of the reads was accomplished using the FastQC tool (https://www.bioinformatics.babraham.ac.uk/projects/fastqc/) and MultiQC tool (https://multiqc.info/) which showed a high quality of sequencing indicated by a GC content peak around 50%, an ambiguity base content of nearly 0%, and an average PHRED score near 40 for all libraries. The Trimmomatic tool (http://www.usadellab.org/cms/?page=trimmomatic) was used (HEADCROP:13, TRAILING:20, SLIDINGWINDOW:4:20, MINLEN:15) to clip Illumina adaptors and trim/remove low quality nucleotides. Intact read pairs were then reference mapped to the Mouse genome (http://daehwankimlab.github.io/hisat2/download/#m-musculus) using HISAT2 (http://daehwankimlab.github.io/hisat2/) under specific conditions (-rna-strandness RF, --fr, --no-discordant, --no-mixed). The returned alignment files were sorted and indexed using samtools (http://www.htslib.org/). Enumeration per gene (Mus_musculus.GRCm38.92.gtf) for each alignment file was accomplished using Stringtie (http://ccb.jhu.edu/software/stringtie/), producing expression in Transcript Per

Million (TPM) units. The TPM values were imported into R (http://www.r-project.org/), pedestalled by 2, Log_2 transformed, and filtered to remove genes not having a transformed value >1 for at least one library/sample. Post-filtering, transformed values for remaining genes were quantile normalized and quality inspected to confirm the absence of outliers via Tukey box plot, covariance-based principal component analysis (PCA) scatter plot, and correlation-based heatmap. Lowess modelling of the normalized data by library class (Coefficient of Variation ~ Mean) was then performed and the fits were plotted. The lowest mean expression value across the fits at which the linear relationship with Coefficient of Variation was grossly lost (value=2) was defined as the noise threshold for the data. Genes not having a value greater than this threshold for at least one library were discarded as noise-biased. Surviving genes having a value less than the threshold were floored to equal the threshold. Statistical comparisons were performed using Analysis of Covariance (ANCOVA) under AIC-step optimization and Benjamini Hochberg (BH) False Discovery Rate (FDR) Multiple Comparison Correction (MCC) condition, correcting for sex. Genes having a p-value < 0.05 and a linear fold difference of adjusted means > = 1.5X were deemed to be different between sample groups. Volcano plots were generated to describe the number, magnitude, and significance of the genes identified. Gene ontology analysis of the dysregulated genes was performed by using the Database for Annotation, Visualization and Integrated Discovery (DAVID) database (https://david.ncifcrf.gov/). Enriched pathways and functions for the dysregulated genes were identified using the Ingenuity Pathway Analysis tool (https://digitalinsights.qiagen.com/).

References:

1. Bonaldo P, Braghetta P, Zanetti M, Piccolo S, Volpin D, Bressan GM. Collagen VI deficiency induces early onset myopathy in the mouse: an animal model for Bethlem myopathy. Hum Mol Genet 1998;7:2135-2140.
2. Pan TC, Zhang RZ, Markova D, et al. COL6A3 protein deficiency in mice leads to muscle and tendon defects similar to human collagen VI congenital muscular dystrophy. J Biol Chem 2013;288:14320-14331.
3. Noguchi S, Ogawa M, Malicdan MC, Nonaka I, Nishino I. Muscle Weakness and Fibrosis Due to Cell Autonomous and Non-cell Autonomous Events in Collagen VI Deficient Congenital Muscular Dystrophy. EBioMedicine 2017;15:193-202.
4. Pan TC, Zhang RZ, Arita M, et al. A mouse model for dominant collagen VI disorders: heterozygous deletion of Col6a3 Exon 16. J Biol Chem 2014;289:10293-10307.
5. de Greef JC, Hamlyn R, Jensen BS, et al. Collagen VI deficiency reduces muscle pathology, but does not improve muscle function, in the gamma-sarcoglycan-null mouse. Hum Mol Genet 2016;25:1357-1369.
6. Smith JW, O'Meally RN, Burke SM, et al. Global Discovery and Temporal Changes of Human Albumin Modifications by Pan-Protein Adductomics: Initial Application to Air Pollution Exposure. J Am Soc Mass Spectrom 2023;34:595-607.
7. Chambers MC, Maclean B, Burke R, et al. A cross-platform toolkit for mass spectrometry and proteomics. Nat Biotechnol 2012;30:918-920.
8. Kessner D, Chambers M, Burke R, Agus D, Mallick P. ProteoWizard: open source software for rapid proteomics tools development. Bioinformatics 2008;24:2534-2536.
9. Kong AT, Leprevost FV, Avtonomov DM, Mellacheruvu D, Nesvizhskii AI. MSFragger: ultrafast and comprehensive peptide identification in mass spectrometry-based proteomics. Nat Methods 2017;14:513-520.
10. Kall L, Canterbury JD, Weston J, Noble WS, MacCoss MJ. Semi-supervised learning for peptide identification from shotgun proteomics datasets. Nat Methods 2007;4:923-925.
11. Nesvizhskii AI, Keller A, Kolker E, Aebersold R. A statistical model for identifying proteins by tandem mass spectrometry. Anal Chem 2003;75:4646-4658.
12. da Veiga Leprevost F, Haynes SE, Avtonomov DM, et al. Philosopher: a versatile toolkit for shotgun proteomics data analysis. Nat Methods 2020;17:869-870.
13. Demichev V, Szyrwiel L, Yu F, et al. dia-PASEF data analysis using FragPipe and DIA-NN for deep proteomics of low sample amounts. Nat Commun 2022;13:3944.
14. Rio DC, Ares M, Jr., Hannon GJ, Nilsen TW. Purification of RNA using TRIzol (TRI reagent). Cold Spring Harb Protoc 2010;2010:pdb prot5439.

Supplementary Figure legends:

Supplementary figure 1. (A) Mendelian ratios from *Col6a2*$^{-/+}$ x *Col6a2*$^{-/+}$ breeders shows a slight reduction of *Col6a2*$^{-/-}$ mice (Male 20.2%, Female 21.0%). **(B)** Rotarod assay fails to show any difference in latency to fall between wildtype, *Col6a2*$^{+/-}$ and *Col6a2*$^{-/-}$ mice. Similar results were obtained in older mice (up to 60 weeks of age) **(C)** Treadmill running assay fails to show any difference in running distance before exhaustion in wildtype, *Col6a2*$^{+/-}$ and *Col6a2*$^{-/-}$ mice. Note the large variability among individual mice at each genotype. **(D)** Activity monitoring does not show a difference in rearing time across wildtype, *Col6a2*$^{+/-}$ and *Col6a2*$^{-/-}$ mice. **(E)** Freewheel running assay fails to show a difference in voluntary running rate across wildtype, *Col6a2*$^{+/-}$ and *Col6a2*$^{-/-}$ mice. Error bars in panel C and D represent SEM.

Supplementary figure 2. (A) Hematoxylin and eosin staining of frozen sections of tibialis anterior (TA), quadriceps (Quad), gastrocnemius (Gastrc), triceps, and diaphragm muscles of wildtype and *Col6a2*$^{-/-}$ mice show dystrophic features with fiber size variability, rare degeneration (arrowhead), and regeneration (arrow) and increased internal nuclei. Scale bar = 50 µm. **(B)** Immunostaining of gastrocnemius muscle for myosin heavy chain subtypes highlighting fiber type I (blue), IIa (green), and IIb (red). Unstained myofibers are considered type IIx. The lower two panels also include wheat germ agglutinin stain (white). There is no qualitative difference or selective loss of specific fiber types in *Col6a2*$^{-/-}$ mouse muscle. **(C)** The myofiber minferet diameter of the entire gastrocnemius muscle sections were quantified and shown in violin plots in 60-week-old mice. Note reduced mean fiber diameter in *Col6a2*$^{-/-}$ mouse muscle and a marked increase in coefficient of variability (CV) consistent with increased number of

atrophic and hypertrophic fibers in *Col6a2*$^{-/-}$ mouse muscle. AU= arbitrary units. Error bars indicated SEM. Statistical comparisons were performed by 2-way ANOVA and Tukey's adjustment for multiple comparisons.

Supplementary figure 3. (A) Maximal twitch force and total tetanic force are markedly reduced in 60-week-old *Col6a2*$^{-/-}$ mouse EDL muscle. The difference in force generation was negligible after normalization to functional cross-sectional area (i.e., specific force). Error bars indicated SEM. Statistical comparisons were performed by 2-way ANOVA and Tukey's adjustment for multiple comparisons. **(B)** Tetanic force declined precipitously after repeated eccentric contractions in male but not female 60-week-old *Col6a2*$^{-/-}$ EDL muscle. Statistical analysis was performed using linear mixed models and Bonferroni adjustment for multiple comparisons. Male: wildtype vs *Col6a2*$^{-/-}$ (p<0.0001); *Col6a2*$^{+/-}$ vs *Col6a2*$^{-/-}$ (p<0.0001). Female: wildtype vs *Col6a2*$^{-/-}$ (p=1.0); *Col6a2*$^{+/-}$ vs *Col6a2*$^{-/-}$ (p=0.216).

Supplementary figure 4 (A) Representative image of Sirius red stain shows increased fibrillar collagen content in *Col6a2*$^{-/-}$ mouse muscle, most notable in animals older 10 weeks and older. Scale bar= 50 μm **(B)** Hydroxyproline content of gastrocnemius muscle is markedly increased in 25 week and 60 week old *Col6a2*$^{-/-}$ animals. **(C)** Quantification of series elastic element, parallel elastic element, and damping coefficient in 10 week and 25 week animals after passive stress-relaxation protocol shows a marked increase in series modulus of elasticity and damping coefficient, but not the parallel elastic element in *Col6a2*$^{-/-}$ EDL muscle. Statistical comparisons were performed by two-way ANOVA and Tukey's adjustment for multiple comparisons (panel B, C). Error bars represent SEM.

Supplementary figure 5. (A) Representative hematoxylin and eosin staining of frozen sections of diaphragm muscles of 60 week wildtype and *Col6a2*$^{-/-}$ mice show dystrophic features with fiber size variability, rare degeneration, and regeneration, increased endomysial fibrosis, and increased internal nuclei. Scale bar = 50 μm. **(B)** Hydroxyproline content of diaphragm muscle is markedly increased in 25 week and 60 week old *Col6a2*$^{-/-}$ animals. **(C)** Thickness of diaphragm muscle is essentially unchanged in 60 week old *Col6a2*$^{-/-}$ animals compared to wildtype controls.

Supplementary figure 6. (A) quantification of TGFβ1, 2 and 3 transcripts in mouse muscle tissue from the RNA-sequencing dataset. **(B)** ELISA based quantification of total TGFβ1 levels in muscle lysates from wildtype, *Col6a2*$^{+/-}$ and *Col6a2*$^{-/-}$ mice shows no marked differences. **(C)** Schematic of the HEK293-luc reporter cell line. In the presence of extracellular TGFβ, TGFβ receptor complex is activated and phosphorylates SMAD2/3 which will subsequently bind SMAD4, translocate to the nucleus, and along with other transcription factors (TF) binds the SMAD-binding element (SBE) promoter and drive luciferase (Luc) transcription. **(D)** HEK293-luc cells have increased luminescence in response to exogenously added recombinant TGFβ (rTGFβ). This response is diminished with neutralizing antibodies against TGFβ1 (TGFβ-Ab Clone 1D11). Conditioned media from *Col6a2*$^{-/-}$ muscle fibroblast cultures results in significantly higher levels of luminescence when added to HEK293-Luc cells compared to wildtype and this response is abrogated by neutralizing antibodies against TGFβ1. The datapoints represent technical replicates (different wells).

Supplementary figure 7: **(A)** Hydroxyproline assay of tibialis anterior (TA) muscle at different timepoints after cardiotoxin injury. **(B)** Native gel electrophoresis and western

blotting of wildtype muscle lysates after cardiotoxin injury shows an increase in collagen VI expression coinciding with reduced active TGFβ-1 levels, most notable at day 7 and 14 after muscle injury. Protein complexes in wildtype muscle lysates after cardiotoxin injury were separated under native conditions and serially probed with antibodies against collagen VI and TGFβ1. During muscle regeneration, collagen VI levels increase, the timing of which corresponds to decreased levels of active TGFβ-1. **(C)** Immunostaining of tibialis anterior muscle sections with collagen VI at different time points after cardiotoxin injury. Scale bar = 200 μm

Supplementary figure 8. (A) Heatmap representation of transcript levels of three latent TGFβ binding proteins (LTBPs) in the mouse RNA-sequencing dataset in this study. Ltbp1, 3 and 4 bind TGFβ while Ltbp2 does not. Ltbp4 is the most abundant Ltbp in wildtype and *Col6a2*$^{-/-}$ mouse muscle tissue. **(B)** Immunofluorescence staining of muscle sections for Ltbp4 and collagen VI. Ltbp4 protein localizes normally to the extracellular matrix in the knock-in *Ltbp4* mouse model or double homozygous *Ltbp4*^{hom}/*Col6a2*$^{-/-}$ mice. Scale bar = 50 μm.

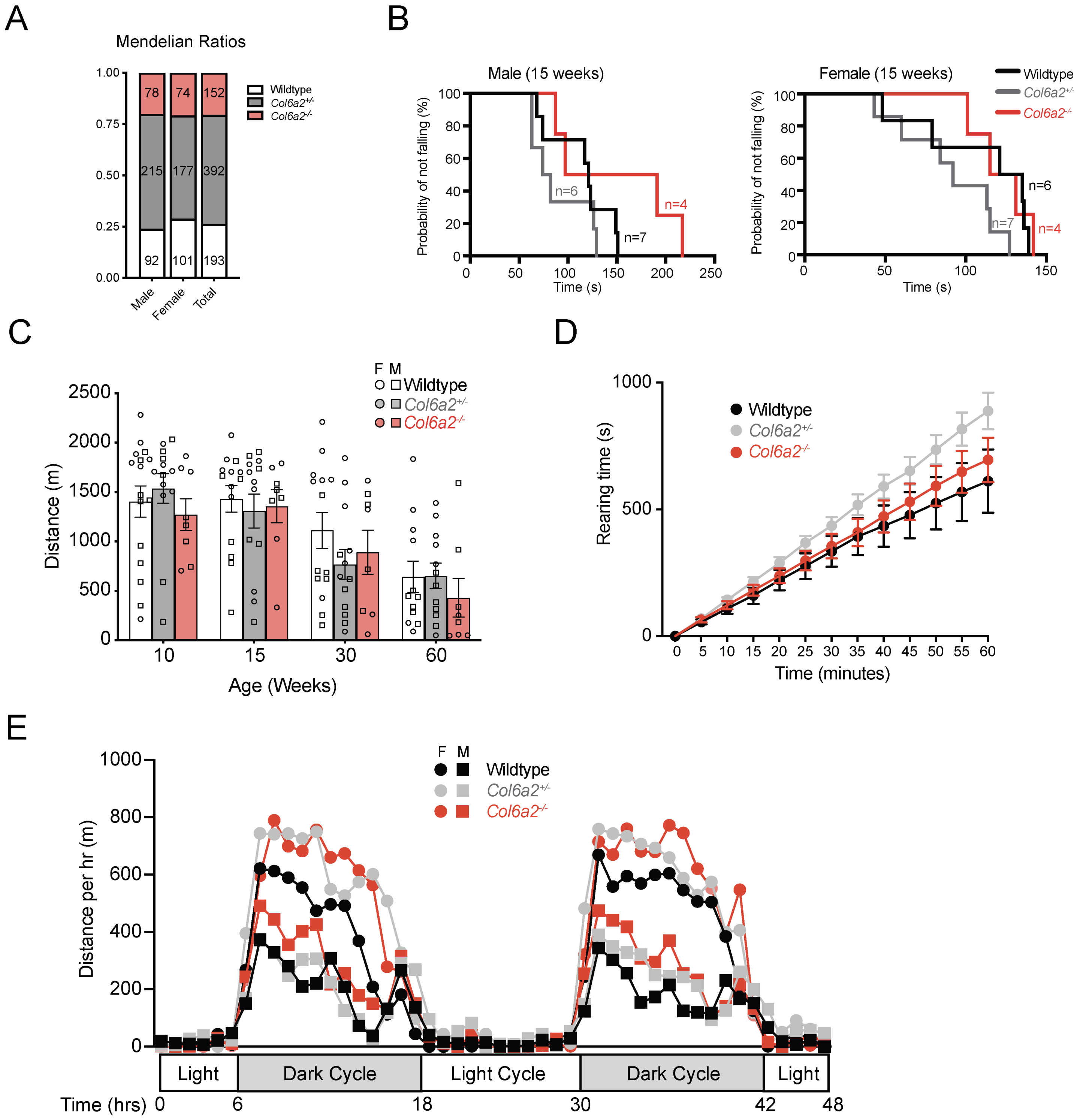

Supplementary figure 1. (A) Mendelian ratios from *Col6a2*$^{+/-}$ x *Col6a2*$^{+/-}$ breeders shows a slight reduction of *Col6a2*$^{-/-}$ mice (Male 20.2%, Female 21.0%). **(B)** Rotarod assay fails to show any difference in latency to fall between wildtype, *Col6a2*$^{+/-}$ and *Col6a2*$^{-/-}$ mice. Similar results were obtained in older mice (up to 60 weeks of age) **(C)** Treadmill running assay fails to show any difference in running distance before exhaustion in wildtype, *Col6a2*$^{+/-}$ and *Col6a2*$^{-/-}$ mice. Note the large variability among individual mice at each genotype. **(D)** Activity monitoring does not show a difference in rearing time across wildtype, *Col6a2*$^{+/-}$ and *Col6a2*$^{-/-}$ mice. **(E)** Freewheel running assay fails to show a difference in voluntary running rate across wildtype, *Col6a2*$^{+/-}$ and *Col6a2*$^{-/-}$ mice. Error bars in panel C and D represent SEM.

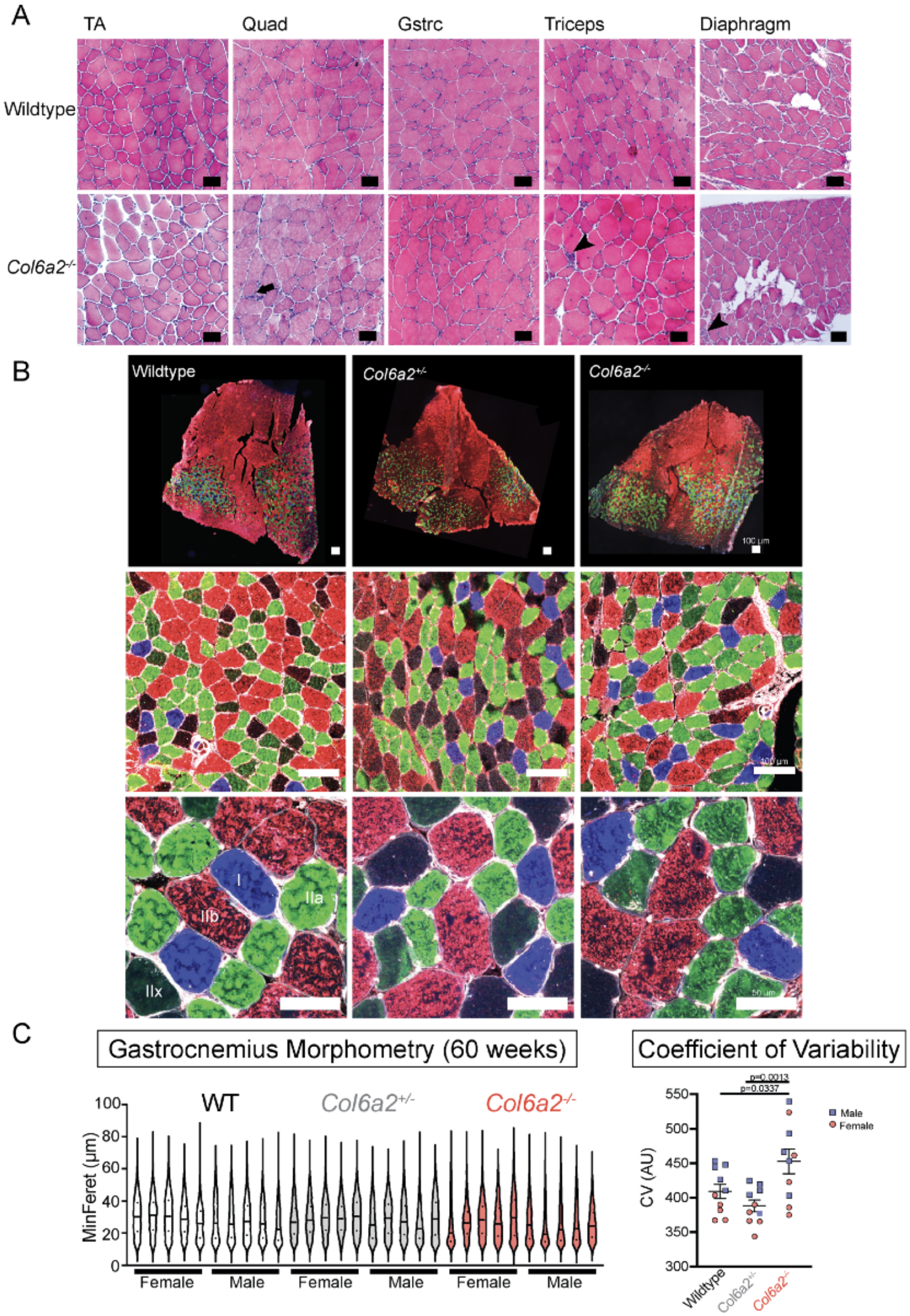

Supplementary figure 2. (A) Hematoxylin and eosin staining of frozen sections of tibialis anterior (TA), quadriceps (Quad), gastrocnemius (Gastrc), triceps, and diaphragm muscles of wildtype and *Col6a2*$^{-/-}$ mice show dystrophic features with fiber size variability, rare degeneration (arrowhead), and regeneration (arrow) and increased internal nuclei. Scale bar = 50 µm. **(B)** Immunostaining of gastrocnemius muscle for myosin heavy chain subtypes highlighting fiber type I (blue), IIa (green), and IIb (red). Unstained myofibers are considered type IIx. The lower two panels also include wheat germ agglutinin stain (white). There is no qualitative difference or selective loss of specific fiber types in *Col6a2*$^{-/-}$ mouse muscle. **(C)** The myofiber minferet diameter of the entire gastrocnemius muscle sections were quantified and shown in violin plots in 60-week-old mice. Note reduced mean fiber diameter in *Col6a2*$^{-/-}$ mouse muscle and a marked increase in coefficient of variability (CV) consistent with increased number of atrophic and hypertrophic fibers in *Col6a2*$^{-/-}$ mouse muscle. AU= arbitrary units. Error bars indicated SEM. Statistical comparisons were performed by 2-way ANOVA and Tukey's adjustment for multiple comparisons.

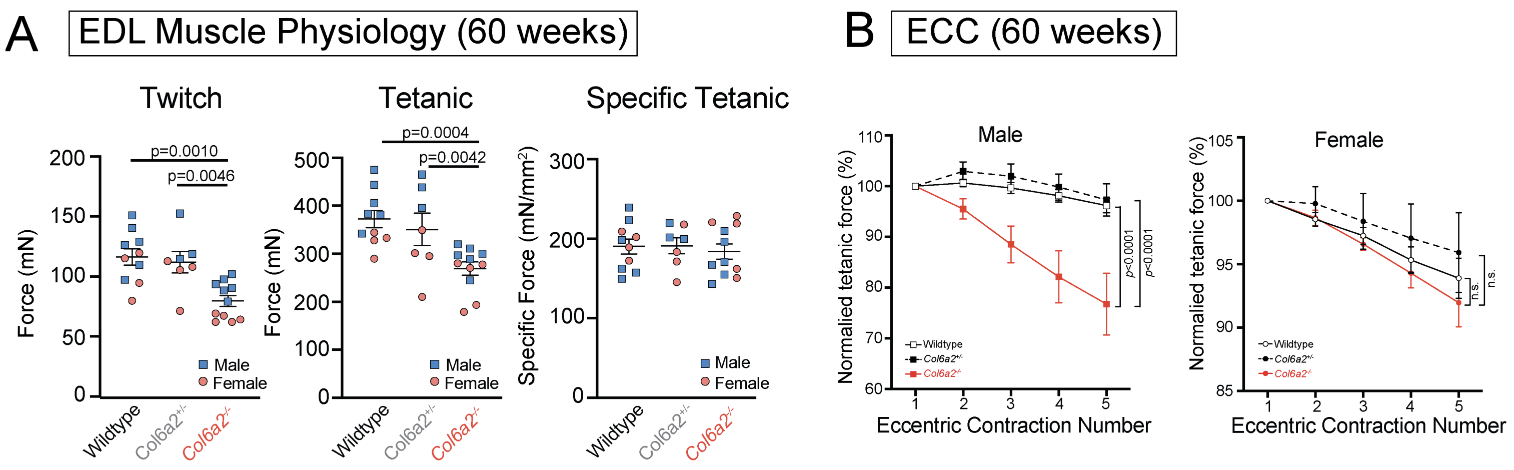

Supplementary figure 3. (A) Maximal twitch force and total tetanic force are markedly reduced in 60-week-old *Col6a2*$^{-/-}$ mouse EDL muscle. The difference in force generation was negligible after normalization to functional cross-sectional area (i.e., specific force). Error bars indicated SEM. Statistical comparisons were performed by 2-way ANOVA and Tukey's adjustment for multiple comparisons. **(B)** Tetanic force declined precipitously after repeated eccentric contractions in male but not female 60-week-old *Col6a2*$^{-/-}$ EDL muscle. Statistical analysis was performed using linear mixed models and Bonferroni adjustment for multiple comparisons. Male: wildtype vs *Col6a2*$^{-/-}$ ($p<0.0001$); *Col6a2*$^{+/-}$ vs *Col6a2*$^{-/-}$ ($p<0.0001$). Female: wildtype vs *Col6a2*$^{-/-}$ (p=1.0); *Col6a2*$^{+/-}$ vs *Col6a2*$^{-/-}$ ($p=0.216$).

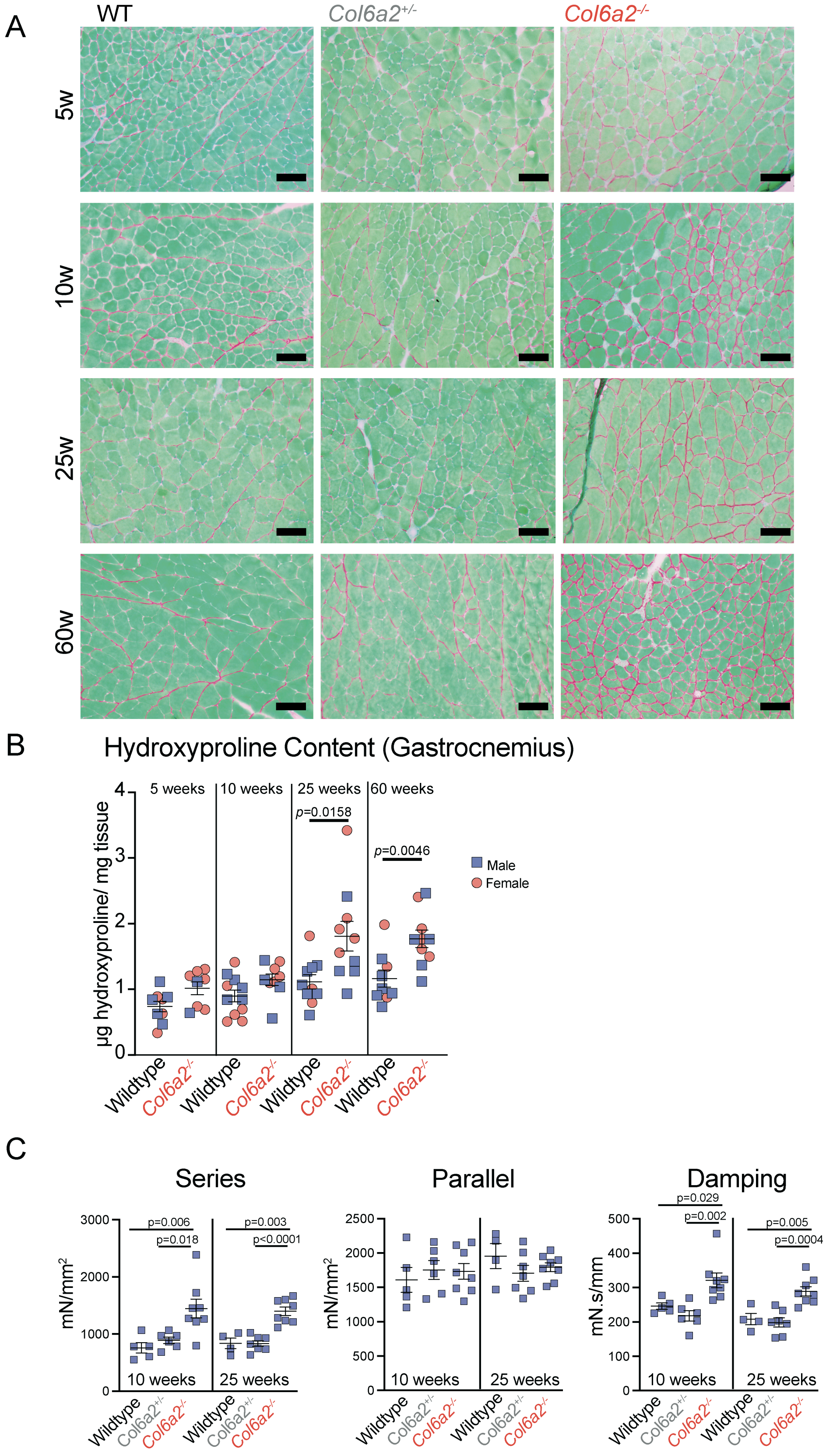

Supplementary figure 4 (A) Representative image of Sirius red stain shows increased fibrillar collagen content in *Col6a2*-/- mouse muscle, most notable in animals older 10 weeks and older. Scale bar= 50 µm **(B)** Hydroxyproline content of gastrocnemius muscle is markedly increased in 25 week and 60 week old *Col6a2*-/- animals. **(C)** Quantification of series elastic element, parallel elastic element, and damping coefficient in 10 week and 25 week animals after passive stress-relaxation protocol shows a marked increase in series modulus of elasticity and damping coefficient, but not the parallel elastic element in *Col6a2*-/- EDL muscle. Statistical comparisons were performed by two-way ANOVA and Tukey's adjustment for multiple comparisons (panel B, C). Error bars represent SEM.

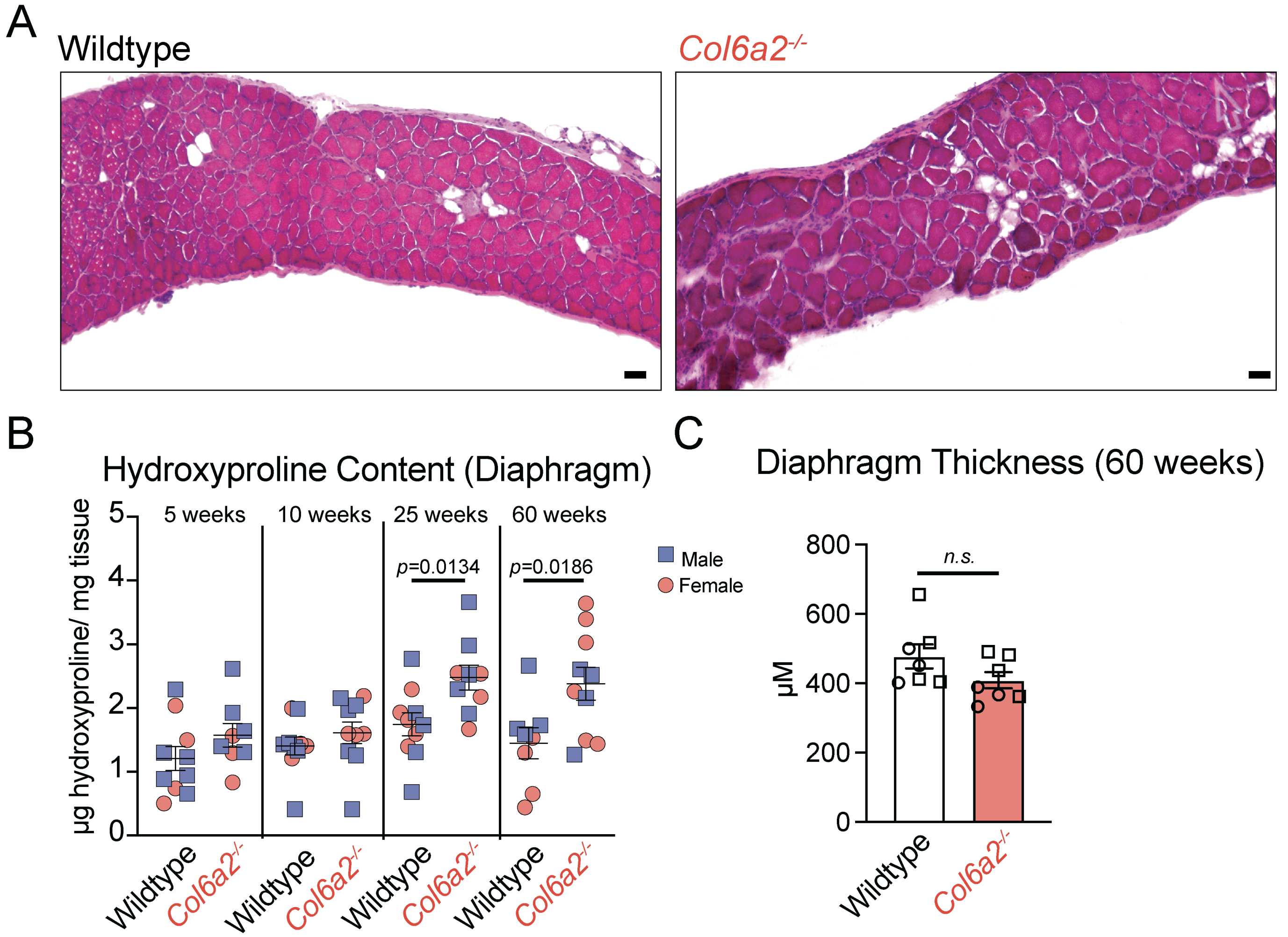

Supplementary figure 5. (A) Representative hematoxylin and eosin staining of frozen sections of diaphragm muscles of 60 week wildtype and *Col6a2*$^{-/-}$ mice show dystrophic features with fiber size variability, rare degeneration, and regeneration, increased endomysial fibrosis, and increased internal nuclei. Scale bar = 50 µm. **(B)** Hydroxyproline content of diaphragm muscle is markedly increased in 25 week and 60 week old *Col6a2*$^{-/-}$ animals. **(C)** Thickness of diaphragm muscle is essentially unchanged in 60 week old *Col6a2*$^{-/-}$ animals compared to wildtype controls.

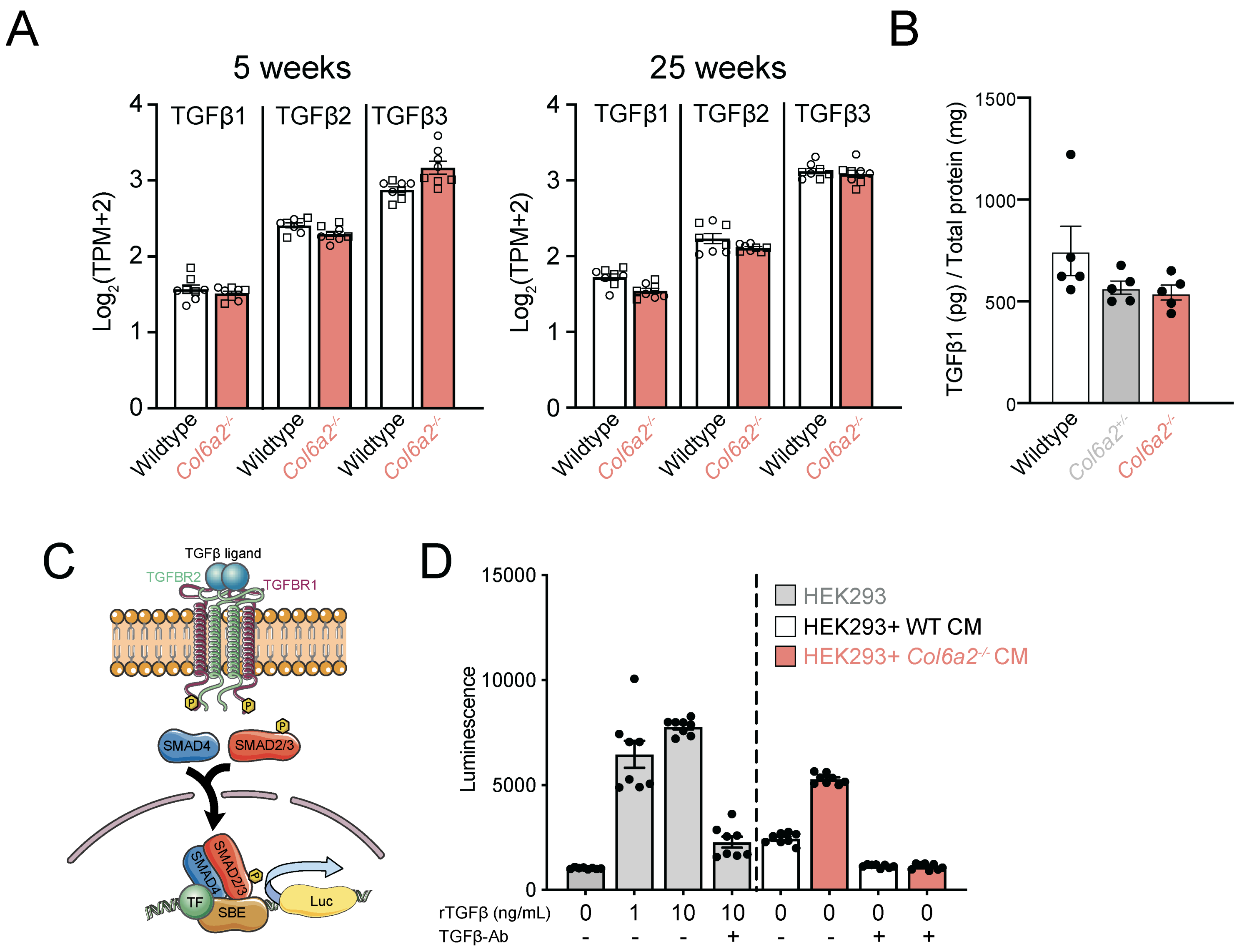

Supplementary figure 6. (A) quantification of TGFβ1, 2 and 3 transcripts in mouse muscle tissue from the RNA-sequencing dataset. **(B)** ELISA based quantification of total TGFβ1 levels in muscle lysates from wildtype, *Col6a2*$^{+/-}$ and *Col6a2*$^{-/-}$ mice shows no marked differences. **(C)** Schematic of the HEK293-luc reporter cell line. In the presence of extracellular TGFβ, TGFβ receptor complex is activated and phosphorylates SMAD2/3 which will subsequently bind SMAD4, translocate to the nucleus, and along with other transcription factors (TF) binds the SMAD-binding element (SBE) promoter and drive luciferase (Luc) transcription. **(D)** HEK293-luc cells have increased luminescence in response to exogenously added recombinant TGFβ (rTGFβ). This response is diminished with neutralizing antibodies against TGFβ1 (TGFβ-Ab Clone 1D11). Conditioned media from *Col6a2*$^{-/-}$ muscle fibroblast cultures results in significantly higher levels of luminescence when added to HEK293-Luc cells compared to wildtype and this response is abrogated by neutralizing antibodies against TGFβ1. The datapoints represent technical replicates (different wells).

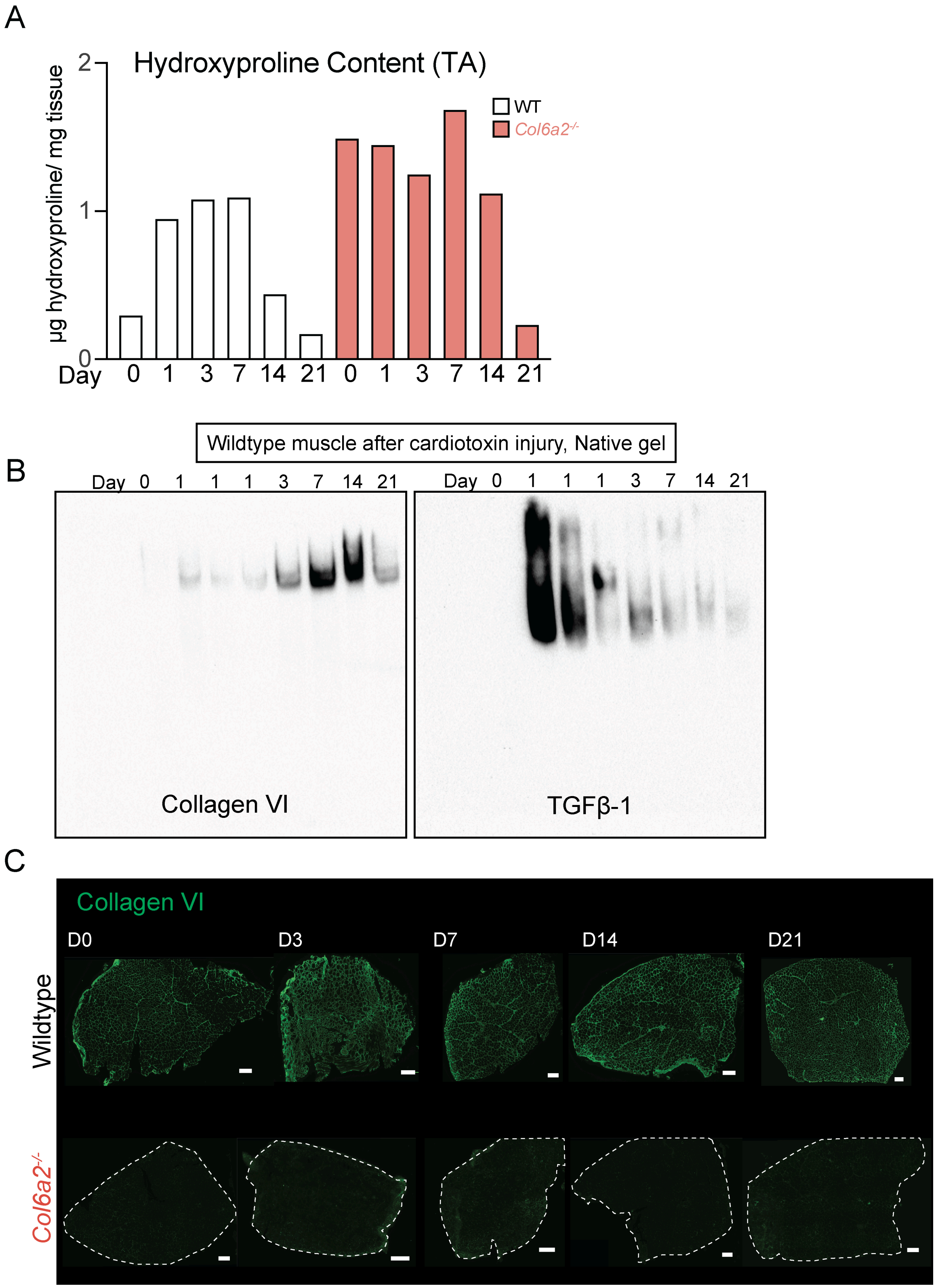

Supplementary figure 7: **(A)** Hydroxyproline assay of tibialis anterior (TA) muscle at different timepoints after cardiotoxin injury. **(B)** Native gel electrophoresis and western blotting of wildtype muscle lysates after cardiotoxin injury shows an increase in collagen VI expression coinciding with reduced active TGFβ-1 levels, most notable at day 7 and 14 after muscle injury. Protein complexes in wildtype muscle lysates after cardiotoxin injury were separated under native conditions and serially probed with antibodies against collagen VI and TGFβ1. During muscle regeneration, collagen VI levels increase, the timing of which corresponds to decreased levels of active TGFβ-1. **(C)** Immunostaining of tibialis anterior muscle sections with collagen VI at different time points after cardiotoxin injury. Scale bar = 200 µm

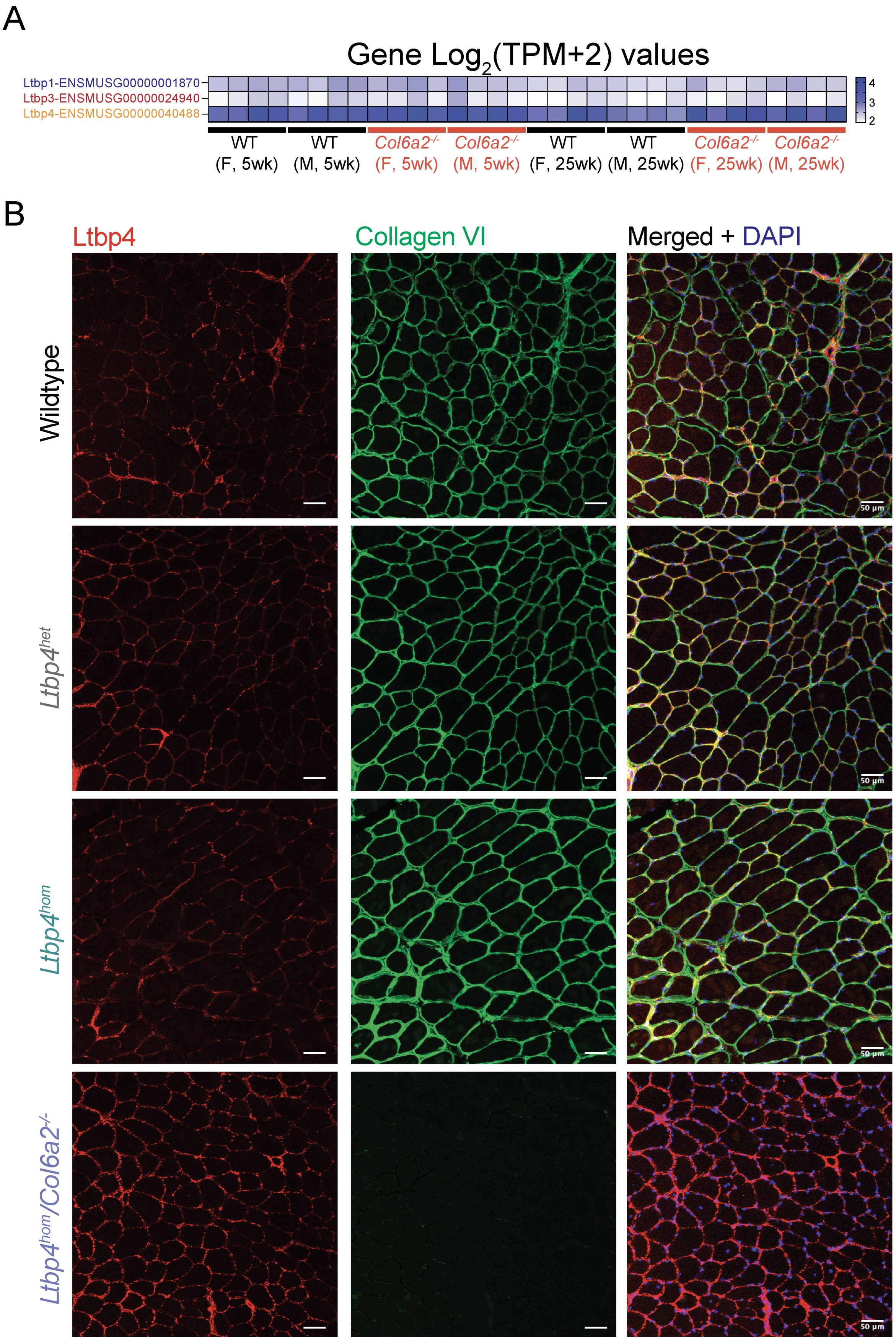

Supplementary figure 8. (A) Heatmap representation of transcript levels of three latent TGFβ binding proteins (LTBPs) in the mouse RNA-sequencing dataset in this study. Ltbp1, 3 and 4 bind TGFβ while Ltbp2 does not. Ltbp4 is the most abundant Ltbp in wildtype and *Col6a2*$^{-/-}$ mouse muscle tissue. **(B)** Immunofluorescence staining of muscle sections for Ltbp4 and collagen VI. Ltbp4 protein localizes normally to the extracellular matrix in the knock-in *Ltbp4* mouse model or double homozygous *Ltbp4*^{hom}/*Col6a2*$^{-/-}$ mice. Scale bar = 50 µm.
