# Supplementary figures and images for: Collagen type VI regulates TGF-β bioavailability in skeletal muscle in mice
Source: J Clin Invest. 2025 May 1;135(9):e173354. doi: 10.1172/JCI173354 (PMC12043086; doi:10.1172/JCI173354)

# Full unedited gels for Figure 6A

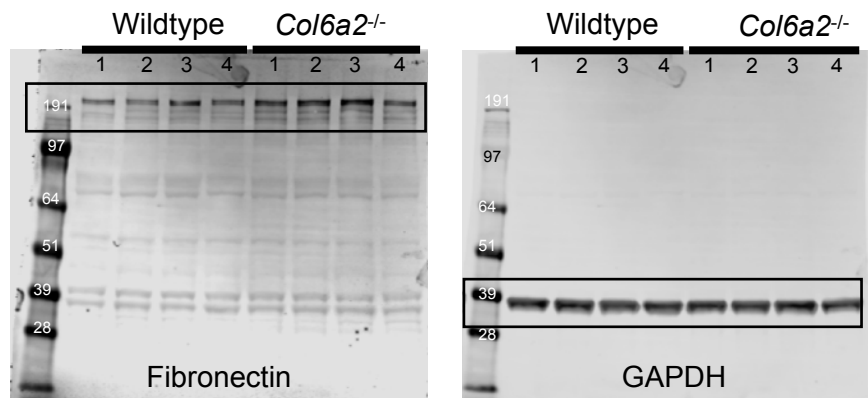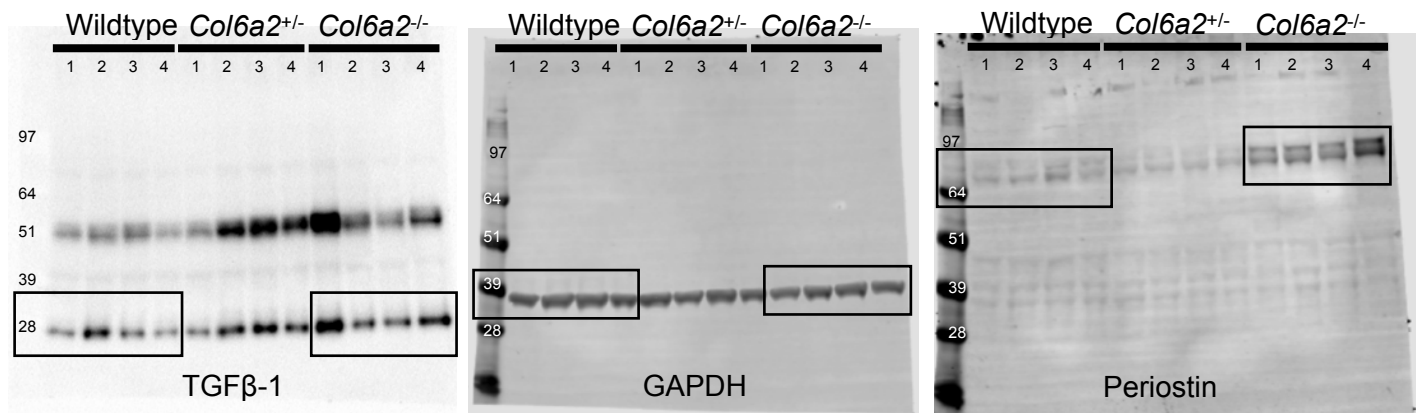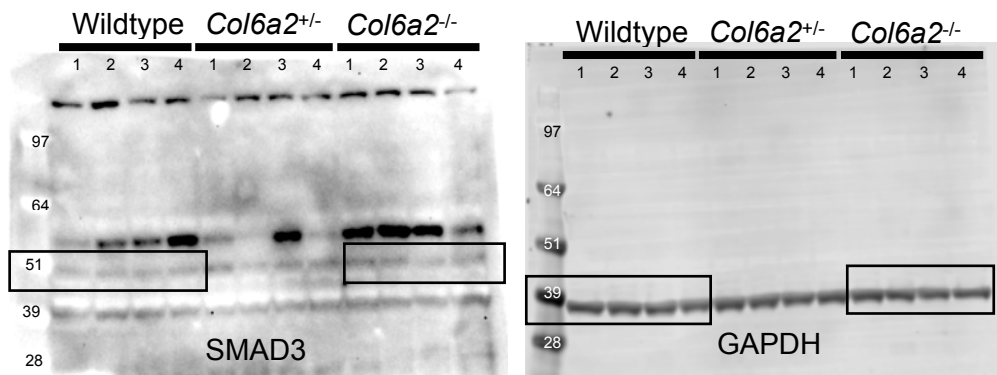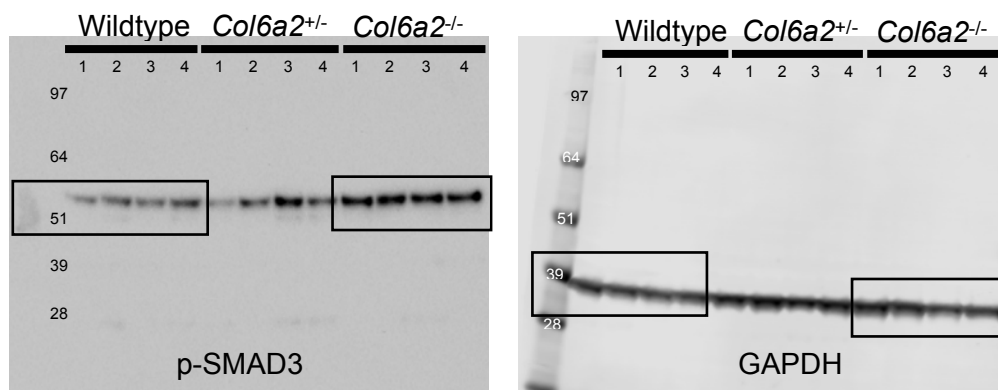

## Full unedited gels for Figure 6D

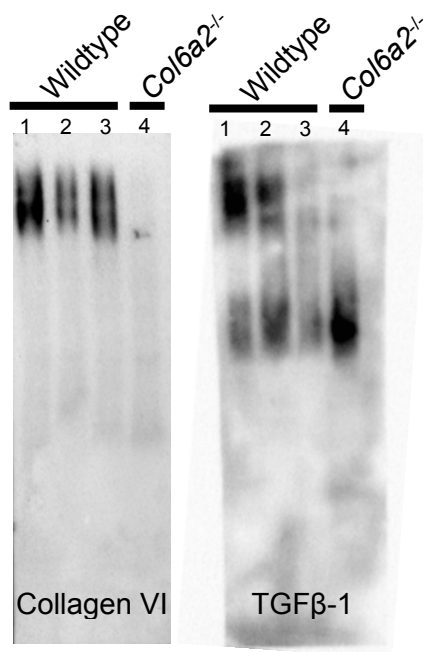

## Full unedited gels for Figure 7D

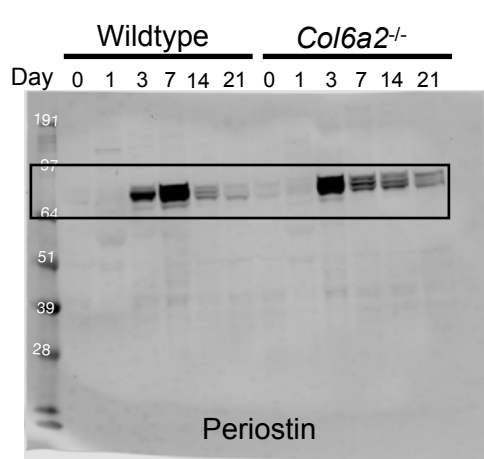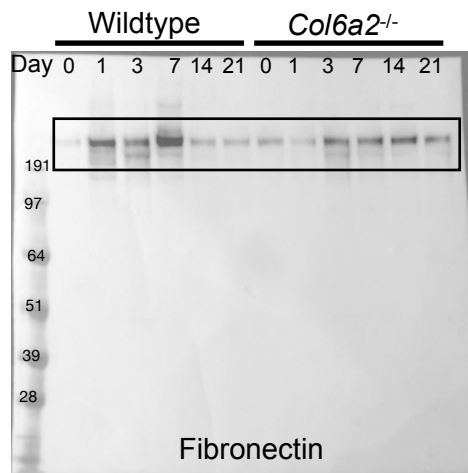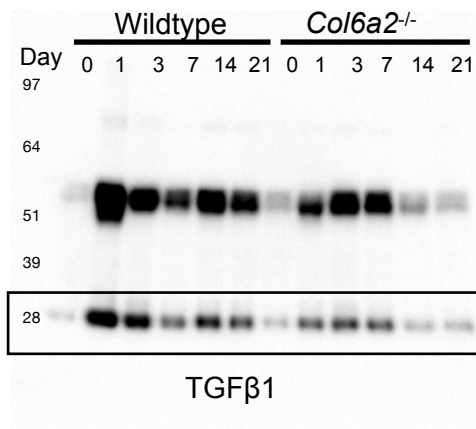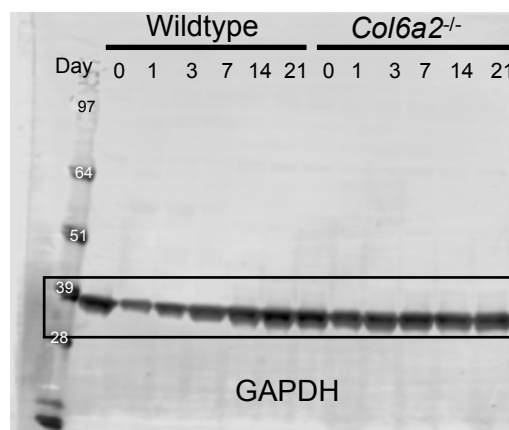

Supplement: Unedited blot and gel images [file jci-135-173354-s271.pdf]
